# Supplementary material for: Convergent Loss of the Necroptosis Pathway in Disparate Mammalian Lineages Shapes Viruses Countermeasures
Source: Front Immunol. 2021 Sep 1;12:747737. doi: 10.3389/fimmu.2021.747737 (PMC8445033; doi:10.3389/fimmu.2021.747737)
Supplement: Supplementary file 1 [file DataSheet_1.pdf]

Supplementary file 1. Accession numbers for *RIPK1*, *RIPK3* and *MLKL* genes found in different mammalian lineages.

Superorder/Order	Superfamily/Family	Species name	*RIPK1*	*RIPK3*	*MLKL*
Primates	Hominidae	*Homo sapiens*	NM 001354930.1	NM_006871.4	NM_152649.4
		Pan troglodytes	XM 016954824.1	XM 001169864.3	XM_016930166.2
		Pan paniscus	XM 003805555.3	XM 003809099.1	XM_024925971.1
	Cercopithecidae	*Papio anubis*	XM 003896962.4	XM 003901654.4	XM_017953558.2
		Macaca mulatta	XM 001091986.3	XM 001114079.3	XM_015126622.1
		M. fascicularis	XM 015449962.1	XM 005560982.2	XM_015443138.1
		Rhinopithecus roxellana	XM 010361800.1	XM 010363712.1	XM_010382960.1
	Cebidae	*Cebus capucinus*	XM 017497471.1	XM 017520641.1	XM_017530561.1
		Aotus nancymaae	XM 021672660.1	XM 012468022.1	XM_012437528.1
		Saimiri boliviensis	XM 010338093.1	XM 003924268.2	XM_010350629.1
Rodents	Muroidea	*Rattus norvegenicus*	NM 001107350.1	NM 139342.1	XM_008772572.2
		Mus musculus	NM 001359997.1	AF178953.1	BC023755.1
	Chinchillidae	*Chinchilla lanigera*	XM 005398899.2	XM 013510393.1	XM_013503862.1
	Caviidae	*Cavia porcellus*	XM 013151465.1	XM 003474285.4	XM_013154995.2
	Octodontidae	*Octodon degus*	XM_004628459.2	**STOP***	**STOP***
	Bathyergidae	*Heterocephalus glaber*	XM_013073264.2	**STOP***	**STOP***
		Fukomys damarensis	XM_010622756.3	**STOP***	**STOP***
	Sciuridae	*Ictidomys tridecemlineatus*	XM 021727876.1	XM 013364463.2	XM_013365168.2
		Marmota marmota	XM 015493957.1	XM 015491924.1	XM_015496076.1
Lagomorpha	Ochotona	*Ochotona princeps*	XM_004596516.1	**INC****	---
		Ochotona curzoniae	---	XM_040969693.1	XM_040982371.1
	Leporidae	*Oryctolagus cuniculus*	XM_017350507.1	**STOP***	---
Chiroptera	Vespertilionidae	*Myotis brandtii*	XM 005869628.2	XM 005859571.2	XM_014536746.1
		Eptesicus fuscus	XM 028136859.1	XM 008158442.2	XM_028142801.1
	Miniopteridae	*Miniopterus natalensis*	XM 016220367.1	XM 016205787.1	XM_016197763.1
	Phyllostomidae	*Desmodus rotundus*	XM 024552320.1	XM 024556248.1	XM_024555929.1
	Hipposidenidae	*Hipposideros armiger*	XM 019668516.1	XM 019663032.1	XM_019646402.1
		Pteropus alecto	XM 025044923.1	XM 006913529.3	XM_025044559.1
		Rousettus aegyptiacus	XM 016166677.1	XM 016120560.1	XM_016143898.1
Carnivora	Mustelidae	*Mustela putorius*	XM 004753625.2	XM 004755192.2	---
		Enhydra lutris	XM 022517756.1	XM 022523405.1	---
	Otoriidae	*Callorhinus ursinus*	XM 025891041.1	XM 025870243.1	---
		Zalophus californianus	XM 027602761.1	XM 027569817.1	---
	Odobenidae	*Odobenus rosmarus*	XM 004408406.2	XM 004402153.1	---
	Ursidae	*Ailuropoda melanoleuca*	XM 002924876.3	XM 011227269.2	---
		Ursus maritimus	XM 008692863.1	XM 008708807.1	---
	Canidae	*Canis lupus*	XM 022414382.1	XM 025442787.1	---
		Vulpes vulpes	XM 025982629.1	XM 026007627.1	---
	Felidae	*Acinonyx jubatus*	XM 027040185.1	XM 015086216.1	---
		Felis catus	XM 023253722.1	XM 003987566.5	---
		Panthera pardus	XM 019419890.1	XM 019429283.1	---
Perissodactyla	Equidae	*Equus asinus*	XM 014867439.1	XM 014868621.1	XM_014850092.1
		Equus caballus	XM 023624365.1	XM 005603254.3	XM_005608429.3

		Ceratotherium simum	XM 004432163.2	XM 004421921.2	XM_014792683.1
**Artiodactyla**	**Bovidae**	*Bos taurus*	NM 001035012.1	XM 005211281.3	XM_024978879.1
		Bubalus bubalis	XM 025264923.1	XM 006061455.1	XM_025268400.1
		Pantholops hodgsonii	XM 005971736.1	XM 005985345.1	XM_005980145.1
		Capra hircus	XM 018038999.1	XM 013966825.2	XM_013970972.2
	**Suidae**	*Sus scrofa*	MG586799.1	XM 001927424.4	MG543991.1
	**Camelidae**	*Camelus dromedarius*	XM 010978020	XM 010995799.1	XM_010987761.1
		Camelus ferus	XM 006175577.2	XM 006172835.2	XM_014554645.1
		Vicugna pacos	XM 006198664.2	XM 006217294.2	XM_015241915.1
**Cetacea**	**Delphinidae**	*Orcinus orca*	XM 004281071.2	**STOP***	**STOP***
		Lagenorhynchus obliquidens	XM 027128236.1	**STOP***	**STOP***
		Tursiops truncatus	XM_019945096.2	**STOP***	**STOP***
		Globicephala melas	XM_030881534.1	**STOP***	**STOP***
	**Phocoenidae**	*Neophocaena asiaeorientalis*	XM_024738443.1	**STOP***	**STOP***
		Phocoena sinus	XM_032650323.1	**STOP***	**STOP***
	**Monodontidae**	*Monodon monoceros*	XM 007452108.1	**STOP***	**STOP***
		Delphinapterus leucas	XM_022586536.2	**STOP***	**STOP***
	**Lipotidae**	*Lipotes vexillifer*	XM 028478546.1	**STOP***	**STOP***
	**Physeteridae**	*Physeter catodon*	XM 028164463.1	**STOP***	**STOP***
	**Balaenopteridae**	*Balaenoptera acutorostrata*	XM 004281071.2	**STOP***	**STOP***
**Afrotheria**	**Elephantidae**	*Loxodonta africana*	XM_010597547.2	**STOP***	**----**
	**Trichechidae**	*Trichechus manatus*	XM_023741740.1	**STOP***	**STOP***
		Elephantulus edwardii	XM 006888715.1	XM 006903266.1	XM_006878861.1
		Echinops telfairi	XM 004711762.1	XM 004698921.1	XM_004704794.1
		Chrysochloris asiatica	XM 006870555.1	XM 006835518.1	**STOP***

* STOP – Presence of an early STOP codon that disrupts the protein.
** INC – Incomplete sequence.
