# Supplementary material for: Convergent Loss of the Necroptosis Pathway in Disparate Mammalian Lineages Shapes Viruses Countermeasures
Source: Front Immunol. 2021 Sep 1;12:747737. doi: 10.3389/fimmu.2021.747737 (PMC8445033; doi:10.3389/fimmu.2021.747737)
Supplement: Supplementary file 2 [file DataSheet_2.pdf]

Supplementary file 2. Accession numbers for *RIPK1*, *RIPK3* and *MLKL* genes found in different Lagomorpha species.

Superorder/Order	Genus	Species name	*RIPK1*	*RIPK3*	*MLKL*
Lagomorpha	*Oryctolagus*	*Oryctolagus cuniculus*	XM_017350507.1	STOP*	---
		Oryctolagus cuniculus algirus	MZ913427	STOP*	---
	Sylvilagus	*Sylvilagus floridanus*	MZ913428	STOP*	---
		Sylvilagus bachmani	MZ913429	STOP*	---
	Lepus	*Lepus americanus*	MZ913430	STOP*	---
		Lepus timidus	MZ913431	STOP*	---
		Lepus europaeus	MZ913432	STOP*	---
		Lepus granatensis	MZ913433	STOP*	---
	Ochotona	*Ochotona collaris*	MZ913434	---	---
		Ochotona princeps	XM_004596516.1	INC**	---

* STOP – Presence of an early STOP codon that disrupts the protein.
** INC – Incomplete sequence.
