# Supplementary material for: Convergent Loss of the Necroptosis Pathway in Disparate Mammalian Lineages Shapes Viruses Countermeasures
Source: Front Immunol. 2021 Sep 1;12:747737. doi: 10.3389/fimmu.2021.747737 (PMC8445033; doi:10.3389/fimmu.2021.747737)
Supplement: Supplementary file 3 [file DataSheet_3.pdf]

Supplementary file 4. Positive and negative selection analyses for RIPK1.

Supplementary file 4.1. Positive selection using SLAC, FEL, MEME and FUBAR methods.

Gene	Amino acids under Positive Selection in the alignment				Total of sites*
	SLAC[a]	FEL[b]	MEME[b]	FUBAR[c]	
RIPK1	183, 204, 258, 293, 294, 465, 501, 517, 518, 537, 539, 541, 543, 552, 689	183, 204, 258, 293, 294, 465, 501, 518, 537, 541, 543, 552, 597, 605, 689	19, 35, 54, 181, 184, 200, 204, 235, 236, 254, 265, 290, 293, 294, 315, 377, 490, 492, 518, 531, 537, 540, 541, 543, 546, 552, 579, 597, 626, 656, 679, 680, 679, 680, 681, 682, 683, 689, 691	204, 293, 294, 518, 543, 689	**10**

a)

Codons with significance level <0.1

b) Codons with significance level <0.05

c) Codons with posterior probabilities >0.90

*) Selected sites in three out of the four methods

Supplementary file 4.2. Negative selection analysis using FEL method with negative residues selected at P > 0.5. Majority of negative selected residues fall in the first 300 aa and at the end of RIPK1 protein.

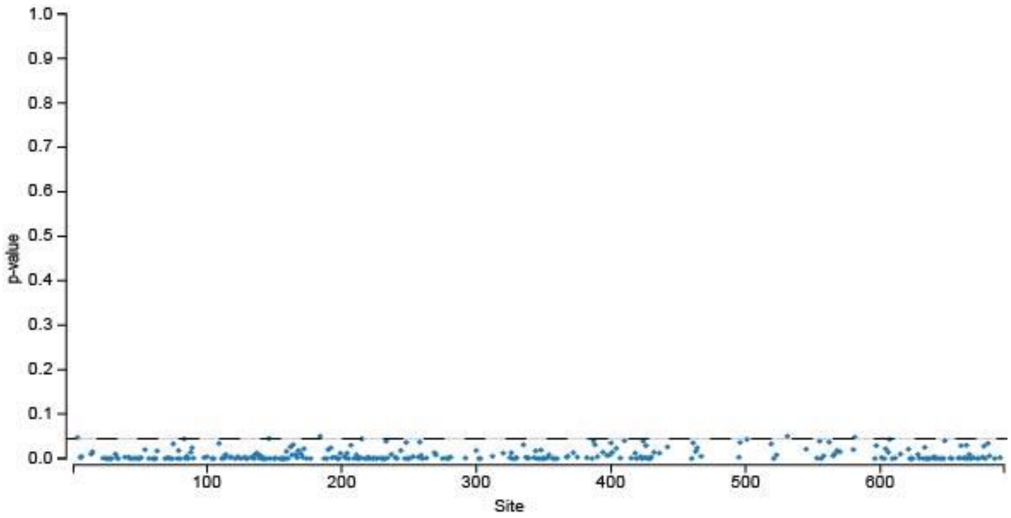

Supplementary file 4.3. RIPK1 protein alignment used for positive and negative analysis.

```
                                     10        20        30        40        50        60        70        80        90       100
                             ....|....|....|....|....|....|....|....|....|....|....|....|....|....|....|....|....|....|....|....|
H. sapiens                   MQPDMSLNVIKMKSSDFLESAELDSGGFGKVSLCFHRTQGLMIMKTVYKGPNCIEHNEALLEEAKMMNRLRHSRVVKLLGVIIEEGKYSLVMEYMEKGNL
P. troglodytes               ....................................................................................................
P. paniscus                  ....................................................................................................
P. anubis                    ........................................FV...........S................................N.............
M. mulatta                   ...................R....................FV......R....S................................N.............
M. fascicularis              ...................R....................FV......R.....................................N.........R...
R. roxellana                 ----.....M.........R.....................V.........S..................................N.............
C. capucinus                 .E..I...A....A.....R.................N...V......T..K.T..S..............................N.............
A. nancymaae                 .E..L...A..........R.................N...V......T..K.T.................................N.............
S. boliviensis               .E..T...A..........R.................N...V......T..K.T.................................N.............
R. norvegicus                .......DN...A.D.L..RKD................H.FV.L.K..T...RA.Y.......G...H....D.......I.....N.........Q...
M. musculus                  .......DN...A...L..KTD............Y..SH.FV.L.K..T...RA.Y..V....G...H............I.....N.............
H. glaber                    ----....D...N...L.QKED.......A....Y...H.FV.L.K..T..KRM.Y..S....G...H..S....MR.........N.............
F. damarensis                ----...DD...N...L.QKED.......A....Y...H.FV.L.K..T..KRM.Y..S....G...Y..S.R..M..........N.............
O. degus                     ----...DD.R.N..EL.WEED.A.....A.FS.Y...YEHV.L.K..T..KRM.Y.......G.V.HQ...RH..R...I.M...N.A....FV...S.
C. lanigera                  ----...ED...N...L.WEED.......A......K.HKHV.L.K..T..KRM.Y..S....G...Y..S.N.....................F......
C. porcellus                 ----...DD...N.A.L.YKRN.......A.......MH..V.L.K..T..KRM.Y..S....G...R..S.........AV..D.N.C...........
I. tridecemlineatus          ----...DD...N...L.KKED................H..V.L.N..T..K.T.Y..S....G.I.H..K.........I.....D.............
M. marmota                   ----...DD...N...L.KKED................H.FV.L.K..T..K.T.Y..S....G.I.H..K.........I.....D.............
O. cuniculus cuniculus       ----...DD..........K.D.......E.......LH..V.L.K..T..KRA.Y..S....GR..H..K.....E....VM...N.C.........S.
O. cuniculus algirus         ----...DD..........K.D.......E.......LH..V.L.K..T..KRA.Y..S....GR..H..K.....E....VM...N.C.........S.
S. floridanus                ----...DD..........K.D.......E.......LH..V.L.K..T..KRA.Y..S....G...Y..K.....E...I.M...N.............
S. bachmanni                 ----...DD..........K.D.......E.......LH..V.L.K..T..KRA.Y..S....G...Y..K.....E...I.M...N.............
L. americanus                ----...DD..........K.D.......E.......LH..V.L.R..T..KRA.Y..S....G...H..K.....E...I.M...N.............
L. timidus                   ----...DD..........K.D.......E.......LH..V.L.R..T..KRA.Y..S....G...H..K.....E...I.M...N.............
L. europaeus                 ----...DD..........K.D.......E.......LH..V.L.R..T..KRA.Y..S....G...H..K.....E...I.M...N.............
L. granatensis               ----...DD..........K.D.......E.......LH..V.L.R..T..KRA.Y..S....G...H..K.....E...I.M...N.............
O. princeps                  ----...DD.......L..KED...............LH..V.L.K..T..KRTDY..S....G...Y..K.....E.......D.N.............
O. collaris                  ----...DD.......L..KED...............LH..V.L.K..T..KRTDY..S....G...Y..K.....E.......D.N.............
M. brandtii                  ----...DD........Q.E.P...............SH..V.L.K..T..KRT.Y.AS....G...H....E.......I.M...N.............
E. fuscus                    ----...DD.........DTEH............L..SH..V.L.K..T..KRT.Y..S....G...H....E.......I.....N.............
M. natalensis                ----...ED.Q..PG.L..REH.......R....L...H..V.L.K..T..K.T.Y..S....G...H....E.......I.L...N...........S.
D. rotundus                  ----...DD..........QEP.C.....Q....L..SY.FV.L.K..T..K.L.Y..S....G...H....E.......L.....N.C...........
H. armiger_                  ----...DD.......V..KED............L..SH.FV.L.K..T..KRT.Y..S....G...H..S.Q.......I.....N.............
P. alecto                    ----...DD..........KEH............L..SH..V.L.K..T..KRT.Y..S....G...HK...Q.......I.....N.............
R. aegyptiacus               ----...DD..........KEF............L..SH..V.L.K..T..KRT.Y..S....G...HK...Q.......I.....N.............
M. putorius                  ----...DD....T..L..KEY............L..CH..V.L.K..T..KRT.Y..S....G...H..................N.............
E. lutris                    ----...DD....T..L..KEY............L..CH..V.L.K..T..KRT.Y..S....G...H....N.............N.............
C. ursinus                   ----...DD..........KEH............L..SH..V.L.K..T..KRT.Y..S....G...H....N.........M...S.............
Z. californianus             ----...DD..........KEH............L..SH..V.L.K..T..KRT.Y..S....G...H....N.........M...S.............
O. rosmarus                  ----...DD..........KEH............L..SH..V.L.K..T..KRT.Y..S....G...H....N.........M...S.............
A. melanoleuca               ----...DD..........KEH............L..SH..V.L.K..T..KRT.Y..S....G...H..................N......F......
U. maritimus                 ----...DD..........KEH............L..SH..V.L.K..T..KRT.Y..S....G...H..................N.............
C. lupus                     ----...DD..........KEH............L..SH..V.L.K..T..KRT.Y..S....GR..H....R.........L...N.............
V. vulpes                    ----...DD..........KEH............L..SH..V.L.K..T..KRT.Y..S....GR..H....R.........L...N.............
A. jubatus                   ----...DD..........KEH............L..SH..V.L.K..T..K.T.Y..S....GR..H....N.........L...N.............
F. catus                     ----...DD..........KEH............L..SH..V.L.K..T..K.T.Y..S....GR..H....N.........L...N.............
P. pardus                    ----...DD..........KEH............L..SH..V.L.K..T..KRT.Y..S....GR..H....N.........L...N.............
E. asinus                    ----...DD.....T....QEY............LN.SHE.V.L.K..T..KRT.YD.S....GR..H....D.......I.....N.A......T....
E. caballus                  ----...DD.....T....QEY............LN.SHE.V.L.K..T..KRT.YD.S....GR..H....D.......I.....N.A......TN...
C. simum                     ----...DD..........KEY............L..SH..V.L.K..T..KRT.Y..S....G...H....N.......I.....N.............
B. taurus                    ----...DD...S.T....Q.D.........C..L..SH..V.L.K..T..KRT.Y.......GR..H....R.........M...S.........Q.D.
B. bubalis                   ----...DD...S.T....Q.D.........C..L..SH..V.L.K..T..KRT.Y.......GR..H....H.........M...S.........Q.D.
P. hodgsonii                 ----...DD...SPT....Q.D.........C..L..SH..V.L.K..T..KRT.Y.......GR..H....C.........M...S.........Q.D.
C. hircus                    ----...DD...SPK....Q.D.........C..L..SH..V.L.K..T..KRT.Y.......GR..H....C.........M...S.........Q.D.
S. scrofa                    ----...DD..........KED.E.............KSH..V.L.K..T..KRN.Y..S....GR..H........R....VM...D....I...A..D.
C. dromedarius               ----...DD.....A....QED.......R....L...H..V.L.K..T..K.T.Y..S....GR..H........Q.....M...S.............
C. ferus                     ----...DD.....A....QED.......R....L...H..V.L.K..T..K.T----------------------------------------------
V. pacos                     ----...DD.....A....QED.......R....L...H..V.L.K..T..K.T.Y..S....GR..H........Q.....M...S.............
O. orca                      ----...DD.R.S.TE..RQED............L.SSHE.VVL.K..T..KRT.YD......GR..H..............M.D.N.......V...D.
L. obliquidens               ----...DD.R.S.TE..KQED............L.SSHE.VVL.K..T..KRT.YD......GR.RH..............M.D.N.......V...D.
T. truncatus                 ----...DD.R.S.TE..KQED............L.SSHE.VVL.K..T..KRT.YD......GR.RH..............M.D.N.......V...D.
G. melas                     ----...DD.R.S.TE..KQED............L.SSHE.VVL.K..T..KRT.YD......GR.RH..............M.D.N.......V...D.
N. asiaeorientalis           ----...DD.R.S.TE..RQED............L.SSH..VVL.K..T..KRT.YD.....DGRT.H..............M.D.N.......V...D.
P. sinus                     ----...DD.R.N.AE..RRED............L.SSH..VVL.K..T..KRT.YD.....DGR..H..............M.D.N.......V...D.
M. monoceros                 ----...DD.R.S.TE..RQED............L.SSH..VVL.K..T..KRT.YD.....DGR..H..............M.D.N.......V...D.
D. leucas                    ----...DD.R.S.TE..RQED............L.SSH..VVL.K..T..KRT.YD.....DGR..H..............M.D.N.......V...D.
L. vexillifer                ----...DD.R.S.TE..RQED..G.........L.SSH..VVL.K..T..KLT.YD......GR..H..............M.D.N.......V...D.
P. catodon                   ----...DD.E.N.T....REG............L..SH..VVL.K..T..KRT.Y.......GR.RH..............M...N.......V...D.
B. musculus                  ----...DD...N.A...LQKD..G.........L..SH..VVL.K..T..RRA.YS......GRV.H........Q.....M.D.N.......L...D.
B. acutorostrata             ----...DD...N.A...QQKD..G.........L..SH..VVL.K..T..KRA.YS......GRV.H..Q.....Q.....M.D.N.......L...D.
L. africana                  ----...DD.....T...QTTH............H..IH..V.L.N..T..K.T.Y..S....G...H..N.N...........D.N.............
T. manatus                   ----...DD.....T...QT.H............C..LH..V.L.N..T..KRT.Y..S....G...H..N.N...........D.N.............
E. edwardii                  ----...DD.E...T.L.VKK.I.N.S..E....L...C..V.L.K..T..K.T.Y..S....G...H..N.N...........D.N.............
E. telfairi                  ....VF.DE.....T...TTRYI..W..........KIHRNV.L.N..T..K...YD.S....G...H..N.H.........M...S........K....
C. asiatica                  ....TC.DD..L..T...NAVH.........C..K.IIL.YV.L.K..T...RA.Y.......GR..H..N.D..M..........N.C......K....

                                    110       120       130       140       150       160       170       180       190       200
                             ....|....|....|....|....|....|....|....|....|....|....|....|....|....|....|....|....|....|....|....|
H. sapiens                   MHVLKAEMSTPLSVKGRIILEIIEGMCYLHGKGVIHKDLKPENILVDNDFHIKIADLGLASFKMWSKLNNEEHNELREV-DGTAKKN-GG-TLYYMAPEH
P. troglodytes               ...............................................................................-....R..-..-.........
P. paniscus                  ...............................................................................-....R..-..-.........
```

```
P. anubis                   ...................V...........................D....................SK......K..-.S.S...-..-.........
M. mulatta                  ...................V...........................D....................SK......K..-...S...-..-.........
M. fascicularis             ...................V...........................D....................SK......K..-...S...-..-.........
R. roxellana                .........I.........V...........................D....................SK......K..-..AS...-..-.........
C. capucinus                ...................V...........................D...............T....SK..........-...S...-..-.........
A. nancymaae                ...................V...........................D...............T....SK..........-.S.S...-..-.........
S. boliviensis              .......V...........V...........................D...............T....SK..........-.S.S...-..-.........
R. norvegicus               .....TKE.V.........V......H...DE...............R..........V....T....TK....KQ..A-SSVT...-..-.........
M. musculus                 .....TQIDV...L.....V.A........D................R..........V....T....TK.KD.KQK..-SS.T...N..-.........
H. glaber                   .Q..R.QI.I.........A...........A..........................V....T....TK.....Q.KA-NS.S...-..-.........
F. damarensis               .Q....Q..I.....A.V.A...........A..........................V....T....TK.....Q.KA-NS.S...-..-.........
O. degus                    ..M...QI.I....R...VA.T.........E.........D.L...K..........V....T....TK.....Q.RA-N...RR.-..-N........
C. lanigera                 .N....QI.I.........A.T.........E..........................V....T....TK.....Q.KA-.S.T...-..-.........
C. porcellus                L...Q.QT-I.........V.T.........E...............S..........V....T..R.TQ.....Q.KA-S..TRN.-..-..H......
I. tridecemlineatus         .Q....QI.I.........V.T.........E...............DN.........V....T....TK.K...Q.K.-NSNSV..-..-.........
M. marmota                  .Q....QI.I.........M.T.........E...............DN.........V....T....TK.K...Q.KM-NSNSV..-..-.........
O. cuniculus cuniculus      .......I.I.........A.T.........E..........................V....T....TK.....Q.K.-KS.R..T-..-..C......
O. cuniculus algirus        .......I.I.........A.T.........E..........................V....T....TK.....Q.K.-NS.R..T-..-..C......
S. floridanus               .......I.I.........A.TV........E...............S..........V....T....TK.....Q.K.-NS.R..S-..-..C......
S. bachmanni                .......I.I.........A.T.........E...............S..........V....T....TK.....Q.K.-NS.C..S-..-..C......
L. americanus               .......I.I........VA.T.........E..........................V....T....TK.....Q.K.-NH.S...-..-.........
L. timidus                  .......I.I........VA.T.........E..........................V....T....TK.....Q.K.-NH.S...-..-.........
L. europaeus                .......I.I.........A.T.........E..........................V....T....TK.....Q.K.-NR.S..S-..-.........
L. granatensis              .......I.I.........A.T.........E..........................V....T....TK.....Q.K.-NR.S..S-..-.........
O. princeps                 .Q....KINI.........A.T.........E..........................V....T....TT.....Q.KA-HSAS...-..-.........
O. collaris                 .Q....KINI.........A.T.........E..........................V....T....TT.....Q.KA-HSAS...-..-.........
M. brandtii                 .......I.I.........M...........E...............DN.........V....T....TK.....Q.KA-.SLS...-..-.........
E. fuscus                   .......I.I.........M.T........RE...............DN.........V....T....TK.....Q.KADSLSK.NG-.--.........
M. natalensis               .......I.I.........M.T.........ESI........................V....T....TKDQ...Q.RA-.SLS...-..-..H......
D. rotundus                 .......I.I...........T....R....E..........................V....T....TK.....Q.KM-NSLSN..-..-.........
H. armiger_                 .......I.I.........M.T........KE.I.............D..........V....T....TK.....Q.K.-NSPK.NG-.T--........
P. alecto                   .....T.S.I.........M.T....R.....N..............D..........V....T....TK..D.KQ.K.-NSSS...-..-.........
R. aegyptiacus              .....T.S.I.........M.T....R.....H..............D..........V....A....TK.....Q.K.-NSPS...-..G.........
M. putorius                 .......V.I...L.....M...........Q.I.............D..........V.........TK.....Q.KL-NRVS...-..-..C......
E. lutris                   .......V.I...L.....M...........Q.I.............D..........V.........TK.....Q.KL-NRVS...-..-..C......
C. ursinus                  .......V.I.........M.T.........Q...............D..........V.........TK.....Q.KL-NSVS...-..-..C......
Z. californianus            .......V.I.........M.T.........Q...............D..........V.........TK.....Q.KL-NSVS...-..-..C......
O. rosmarus                 .......V.I.........M.T.........Q...............D..........V.........TK.....Q.KL-NSVS...-..-..C......
A. melanoleuca              .......V.I.........M.T.........Q...............D..........V.T.......TK.....Q.KL-NSVS...-..-..C......
U. maritimus                .......V.I.........M.TV........Q...............D..........V.........TK.....Q.KL-NSVS...-..-..C......
C. lupus                    .......V.I.........M.T.........E...............D..........V....T....TK..I..Q.KL-NSVS...-..-..C......
V. vulpes                   .......V.I.........M.T.........E...............D..........V....T....TK..I..Q.KL-NSVS...-..-..C......
A. jubatus                  .......V.I.........M.T.........E...............D..........V....T....TK..N..Q.KMNNSAP...-..-..C......
F. catus                    .......I.I.........M.T.........E...............D..........V....T....TK..N..Q.KMNNSAP...-..-..C......
P. pardus                   .......I.I.........M.T.........E...............D..........V....T....TK..N..Q.KMNNSAPE..-..-..C......
E. asinus                   .R.....I.I.........M.T.........E...............G..........V.........TK..N..Q.KL-NS.S...-..-..C......
E. caballus                 .R.....I.I.........M.T.........E...............G..........V....T....TK.....Q.KL-NS.S...-..-..C......
C. simum                    .R.....I.I.........M.T.........E...............D..........V....T....TK.....R.K.-NR.S...-..-..C......
B. taurus                   .R....QV.I.........M.T....R....E...............S..........V....T....TK.....Q.KA-G.S.G.S-..-..H......
B. bubalis                  .R....QV.I.........M.T....R....E...............S..........V....T....TK.....Q.KA-G.S.G.S-..-..H......
P. hodgsonii                .R....QV.I.......V.M.T....R....E...............S..........V....T....TK..R..Q.KA-G.G--.S-..-..H......
C. hircus                   .R....QV.I.......V.M.T....R....E...............S..........V....T....TK.....Q.KA-G.S.G.S-..-..H......
S. scrofa                   .R....QTPV........VM.T....R....E...............D..........V....T....TK.....Q.K.-TS.....-..-..H......
C. dromedarius              .....TQI.I.....A...M.T.........E...............S..........V.........TK.....EKKA-SSASQ..-..-..H......
C. ferus                    -----------V...A...M.T.........E...............S..........V.........TK.....EKKA-SSASQ..-..-..H......
V. pacos                    ......QI.I.........M.T.........E...............S..........V.........TK.....EKKA-SS.SQ..-..-..H......
O. orca                     .RA...QI.I.........M.T....R....E...............G.............L.T..E.MK..LTAQ.RG-NS.T.N--..-..H......
L. obliquidens              .RA...QI.I.........M.T....R....E...............G.............L.T..E.MK..LTAQ.RG-NS.T.N--..-..H......
T. truncatus                .RA...QI.I.........M.T....R....E...............G.............L.T..E.MR..LTAQ.RG-NS.T.N--..-..H......
G. melas                    .RA...QI.I.........M.T....R....E...............G.............L.T..E.MK..LTAQ.RG-NS.T.N--..-..H......
N. asiaeorientalis          .RA...QI.I.........M.T....R....E.............I.G.............L.T..E.MK..LTAQ.RG-NS.T.N--..-..H.V....
P. sinus                    .RA...QI.I.........M.T....R....E.............I.G.............L.T..E.MK..LTAQ.RG-NS.T.N--..-..H......
M. monoceros                .RA...QI.I.........M.T....R....E.............I.G.............L.T..E.MK..LTAQ.RG-NS.T.N--..-..H......
D. leucas                   .RA...QI.I.........M.T....R....E.............I.G.............L.T..E.MK..LTAQ.RG-NS.T.N--..-..H......
L. vexillifer               .RT...QI.I.........M.T....R....E...............G.............L.T..E.MK..LTAQ.RG-KS.T.N--.S-..H.....C
P. catodon                  T.....QI.I.........M.T....R...KE.............L.G..........F..L.T.NE.MK..LTAQ.RG-NSST.S--..-..H......
B. musculus                 ......QI.I.........M.T........RE.............M.G...V......F..L.T..E.MR..LTAQ.RG-NS.T.D--..-S.H.T....
B. acutorostrata            ......QI.I.........M.T........RE.............M.G..........F..L.T..E.MR..LAAQ.R--GNSTT.D-S.-S.H.T....
L. africana                 .Q...G.V.I...........T.........E.L.............D..........V....T....TK.....Q.K.-NC.S.R.-..-.........
T. manatus                  .Q...G.V.I...........TV........E...............D..........V....T....TK.....Q.K.-NC.S.R.-..-..C......
E. edwardii                 .R.....I.I...........T........RE.I..............Y.........V.T..T....TK.....Q.KQ-TFISRR.-A.-..C......
E. telfairi                 .Q...W.I.I...........T.........E...............D..........V.........TK..R..Q.KM-NSAS.SS-..-..C......
C. asiatica                 .Q..TDKVRI...........T.........A...............D..........V....T....TK....LQ.KM-TTPS...-..-.VC.I....

                                    210       220       230       240       250       260       270       280       290       300
                            ....|....|....|....|....|....|....|....|....|....|....|....|....|....|....|....|....|....|....|....|
H. sapiens                  LNDVNAKPTEKSDVYSFAVVLWAIFANKEPYENAICEQQLIMCIKSGNRPDVDDITEYCPREIISLMKLCWEANPEARPTFPGIEEKFRPFYLSQLEESV
P. troglodytes              .............................................................................................N......
P. paniscus                 ....................................................................................................
P. anubis                   .....................................................A.I.H..........................................
M. mulatta                  .......................................................I............................................
M. fascicularis             .......................................................I............................................
R. roxellana                .........................................V...........E.I.................D..........................
C. capucinus                ......R...........I...V...H.................I..........I..................L.V.........T.............
A. nancymaae                ...I..............I...V............................L...I.H.........Q......L.V.........E.............
S. boliviensis              ......R...........I...V................................I..................L.V.....................R.
R. norvegicus               .T.I.T............I..............V..TE.FLV..N.....N.E..L.F.........ER..QT...D....F...KE.K......F..Y.
M. musculus                 ...I.............GI.......K......V..TE.FVI........N.EE.L...........ER..Q.I..D....L....E.......HF..Y.
```

```
H. glaber               .S.I.VR.........GI...............SAEH.LI..RT....N.EE...R..K...H..E....ED..V.....S...Q.K...E.E...F.
F. damarensis           .CNI.VR.........GI...............SAEH.LI..RN....N.EE.I.H..K...H..E....E...V..V..S...Q.K...E.EF....
O. degus                .SNI.I..........GI.............T..VE..LL...G....N..E...R..KGV.D......RED..L.......D.Q.K.I...EF..F.
C. lanigera             .SNI.V..........GI.............T..VE...V...R.D...M.E.I.H..K...D..Q...KEE..L.......D....YK...V.E...F.
C. porcellus            .C.I.V..........GIM....I.D.....Y.LSAEH.LV...R.......K.I.H..T......TC..QED..L.........Q.K...E..F..F.
I. tridecemlineatus     ...I............GI................AE..LI.......N.E..I.H..K......E........V.....S.........V..F..Y.
M. marmota              ...I............GI................AE..LI.......N.E..I.H..K......E....T...V.....S.........V..F..N.
O. cuniculus cuniculus  ..................I....VL...............L...........VA.D..G.L.R..E.....D.R......D...R.....S.HY..F.
O. cuniculus algirus    ..................I....VL...............L...........VA.D..G.L.R..E.....D.RV.....D...R.....S.HY..F.
S. floridanus           ..................I......................L............A.D..G.VVN..E....D.D.RV.....D...R.....S.HY..Y.
S. bachmanni            ..................I......................L............A.D..G.VVN..E.....D.RV.....D...R.....S.HY..Y.
L. americanus           ...I..............I......................L.......N....A.D..G.VVG..E.....D.R......D.........S.HY..Y.
L. timidus              ...I..............I......................L.......N....A.D..G.VVG..E.....D.R......D.........S.HY..Y.
L. europaeus            ...I..............I......................L.......N....A.D..G.VVG..E.....D.R...M..D.........S.HY..Y.
L. granatensis          ...I..............I......................L.......N....A.D..G.VVG..E.....D.R...M..D.........S.HY..Y.
O. princeps             ..................I......................L..........C.I.H......G..Q...NED.R......D..GN.K...S.N...N.
O. collaris             ..................I......................L..........C.I.H......G..Q...NED.R......D..GN.K...S.N...N.
M. brandtii             ...I..R.S.........I...............N....LI.......N.N...I....K.......Q....D..V....R....Q.......HN...DI
E. fuscus               ...I..R.S.........I......................I........H....I.F..K.......Q....D.GV.........I.......HN..DD.
M. natalensis           .DN...R.SD........I.......H..............I...A....N.N...I....K.......Q..DPD.QV....A...........HN...C.
D. rotundus             ...I..R.S.........I...............T....V..........EG..I....K.......Q..V.D..L....A...........H..K.D.
H. armiger_             ...I..R.S.........I..............................N.E..I....K.....I..E....T...V........D......S.....F.
P. alecto               ...I..R.S.........I.......H......................E..I.N..K...NI..Q.......V....A....N..........N.
R. aegyptiacus          ...I..R.S.........I.......H......................E..I.N..K...NI..Q.......V....A....E..........N.
M. putorius             ...I....S.......GI.................E...I.......N.E..L....E....I..Q....K.DI....I.L........VD....N.
E. lutris               ...I....S.......GI.................E...I.......N.E..L....E....I..Q....K.DI....T.L........VD....N.
C. ursinus              ...I....S.......GI.............................N....L....V....I..Q..DTK..V....T..........VV....N.
Z. californianus        ...I....S.......GI.............................N....L....V....I..Q..DTK..V....T..........VV....N.
O. rosmarus             ...I....S.......GI.............................N....L....V....I..Q..DMK..V....T..........VV....N.
A. melanoleuca          ...I.T..S.......GI.............................N.E..L....E....I..Q....K.......T..........VD....N.
U. maritimus            ...I....S.......GI.............................N.G..L....E....I..Q....R.......T..........VD....N.
C. lupus                ...I....S.......GI.......................L.........E..I....E....I..Q....K..I....A..........ED....N.
V. vulpes               ...I....S.......GI.......................L.........E..I....E...GI..Q....K..I....A..........ED....N.
A. jubatus              ...I....S.......GI.......................I.......N.E..I....E....I..Q....D..V....A..........VD....N.
F. catus                ...I....S.......GI.......................I.......N.E..I....E....I..Q....D..V....A..........VD....N.
P. pardus               ...I....S.......GI.......................I.......N.E..I....E....I..Q....D..V....A..........VD....N.
E. asinus               .......S.........I.......................I.......N.E..I....K.......H.......V....A....N......D....N.
E. caballus             .......S.........I.......................I.......N.E..I....K.......H.......V....A....N......D....N.
C. simum                .......S.........I.......................I.........E..I....K.......Q.......V....V....N.....VN....N.
B. taurus               .....SR.S.....F...I.......................L..........E..I.F....V.DI.RQ...V..DN....A..........SN...DY.
B. bubalis              .....SR.S.....F...I.......................L..........E..I.F....V.DI.RQ...G..DN....A.......H..SN...DY.
P. hodgsonii            .....SR.S.....F...I.......................L..........E..I.F....V.D..RQ...V..DD....V..........SN...NY.
C. hircus               .....SR.S.....F...I.......................L..........E..I.F....V.D..RQ...V..DD....A..........SN...NF.
S. scrofa               ......R.S.........I.......................L.......S....V.F....VVG...Q..ALS.DD....A.....Y.........GN.
C. dromedarius          ........S.........I.......................V.......S.G..I.H....V.GI..Q..DV....D....A..........SN....N.
C. ferus                ........S.........I.......................V.......S.G..I.H....V.GI..Q..DV....D....A..........SN....N.
V. pacos                ........S.........I.......................V.......S.G..I.H....V.GI..Q..DV....D....A..........SN....N.
O. orca                 ....SSR.S.....F...I....V.........V.......R....S....E...GH....AV.I.EQ..AV...D....A..........N....H.
L. obliquidens          ....SSR.S.....F...I....V.........V.......R....S....E...GH..W.AV.I.EQ..AV...D....A..........N....H.
T. truncatus            ....SSR.S.....F...I....V.........V.......R....S....E...GH....AV.I.EQ..AV...D....A..........N....H.
G. melas                ....SSR.S.....F...I....V.........V.......R....S....E...GH....AV.I.EQ..AV...D....A..........N....H.
N. asiaeorientalis      ....SSR.S.....F...I....V.........P.......Y.....S...TE...GH....AV.I..Q..AV...D....A..........N....H.
P. sinus                ....SSR.S.....F...I....V.........P.......Y.....S...ME...GH....AV.I..Q..AV...D....A..........N....H.
M. monoceros            ....SSR.S.....F...I....V.........P......L.....G..G.E...GQ....AV.I..Q..AV...D....A..........N....H.
D. leucas               ....SSR.S.....F...I....V.........P......L.....G....E...GQ....AV.I..Q..AV...D....AC.........N....H.
L. vexillifer           ....SSR.S.....F...I....V..................R....S....E...GH....AV.I.EQ..AV...D....A....R......N....H.
P. catodon              ...A.SR.S.....F...M....V........G.......T.H.......A.E.V..Q..Q.A..I..Q..AV...D....A..........N....H.
B. musculus             ....SSR.S.....F...I.....................V..RV...D....E.LA.H....A..I..Q..AVD.DD....A......K....N...GH.
B. acutorostrata        ....SSR.S.....F...I....V..................RV...D....E.LA.H....A..I..Q..AVH.DD....A......K....N....H.
L. africana             ..................I......................L.......N.E..I.H..E.......H....S..V....A....NYK....K...KN.
T. manatus              ..................I......................I.......N.E..I.H..K.......H.......V....A....N.KD....E...KD.
E. edwardii             .D.I..............I.......H.......MN.....A....N.....H.N..I.H..K.......RQ...SK.....P..A....T.KS.......DK.
E. telfairi             .....V............I......................I..T.....K.E..I....K...A....Q...A....V....A....N...S...KE...N.
C. asiatica             .S................I.............H.........I.......S....VK...Q...D...H............A.......D.KS...KE...N.

                                 310       320       330       340       350       360       370       380       390       400
                        ....|....|....|....|....|....|....|....|....|....|....|....|....|....|....|....|....|....|....|....|
H. sapiens              EEDVKSLKKEYSN--ENAVVKRMQSLQLDCVAVPSSRSNSATEQPGSLHSSQGLGMGPVEESW---FAPSLEHPQ-EENEPSLQSKLQDEANYHLYGSRM
P. troglodytes          .............--................................................---.........-........................
P. paniscus             .............--................................................---.........-........................
P. anubis               .............--Q.................P.............................---.......Q.-...........E............
M. mulatta              .............--Q..............P..P.............................---.......Q.-...........E............
M. fascicularis         .............--Q..............P..P.............................---.......Q.-...........E............
R. roxellana            .............--Q.................P.............................---.......R.-...........E............
C. capucinus            .............--..............L.P..................F.I...........---.......Q.-...V....N...E.T.........
A. nancymaae            .............--..............L.P..................F.I...........---....V..Q.-...V....N...E.T.........
S. boliviensis          .............--..............L.P..................F.V...........---.......Q.-...V....N...E.T.........
R. norvegicus           ....A......PS--QSP.L...F....H....PL.P.....--...........P........---.SS.P.Y..-....R.V.A....E..S...AF.IFA
M. musculus             ....A......PD--QSP.LQ..F....H....PL.P.....--...........Q........---.SS.P.Y..-D...DR.V.A....E..S...AF.IFA
H. glaber               .K.L.......PM--Q.E.LR..........E.P.....--...........EI.......---.SA....Q.-..H..I..H....E.....VF....
F. damarensis           .K.........PM--Q.E.FR.....R.....D.P.....--...........E........---.SA.P.--.E..H.SN..C....E......I.....
O. degus                .K...E....D.PI--QKE.LR.RH...P...PE.P.....--...........EI......D---.SA....Q.-..Y..I..H....K..D...AF.R..
C. lanigera             .K......E..PI--Q.EILR.VP.V.P...PE.P.....--...........PE........---.SA....Q.-..Y..I..R....K.....VF....
C. porcellus            .K.......K.PT--K.EALM.A.........PE.L.....--..........P..T......Y---.SA.M.RQ.-..Y..I..H....E......F...I
I. tridecemlineatus     ....E......PM--K...LQ.L.........E.P.....--...........QI........---.SA....N..-..S..I..L....E..S...AF.N..
M. marmota              ....E......PM--K...LQ.L.........E.P.....--...........QI........---.S.....N..-.DS..I..L....E..S...AF.N..
O. cuniculus cuniculus  .........R.PE--QS..............P..P.........S.......FMT....V.F---.SACP.IS.-.............E..........
O. cuniculus algirus    .........R.PE--QS..............P..P.........S.......FMT....V.F---.SACP.IS.-.............E..........
```

```
S. floridanus            .........R.PE--QS..............P..P..........S.......FMT....V.F---.SACP.IS.-............E...........
S. bachmanni             .........R.PE--QS..............P..P..........S.......FMT....V.F---.SACP.IS.-............E...........
L. americanus            ........RQ.PE--QS..............PA.P..........S.......FMT....V.F---.SACP.IS.-............E...........
L. timidus               ........RQ.PE--QS..............PA.P..........S.......FMT....V.F---.SACP.IS.-............E...........
L. europaeus             ........RQ.PE--QS..............PA.P..........S.......FMT....V.F---.SACP.IS.-............E...........
L. granatensis           ........RQ.PE--QS..............PA.P..........S.......FMT....V.F---.SACP.IS.-............E...........
O. princeps              .........D.PE--V...............PA.A..........N.......FQT....MTFASL.SA.P..L.-...Q........E...........
O. collaris              .........D.PE--V...............PA.A..........N.......FQT....MTFASL.SA.P..L.-...Q........E...........
M. brandtii              ....Q......PS--P.E.........I....I.P..........R......S.RI.......---....PDYQ.-....R...N...E...........
E. fuscus                ...........PS--Q.E.........I......P......ID..S........R........---....PDYQP-....Y...N...E......F....
M. natalensis            ..........FPS--Q.E.........I....I.P..........S........RI.......---....PD.Q.-.QS.LI..N...E......F....
D. rotundus              ...........PG--Q.EI........M....I.P..........C.................---....P..Q.-...DLR......E......F.N..
H. armiger_              ...........PC--Q.EM........M....I.P............................---.D..P..Q.-........N..........F....
P. alecto                ...........PS--Q.EI........M....I.P..........S........RI.......---....P..Q.-....L.......E......F....
R. aegyptiacus           ...........PS--Q.EI........M....I.P..........S........RI.......---....P..Q.-....L.......E......F....
M. putorius              .D..T....Q.PDPDQSSF....H...I..T.I.P......P...S.........I.......---......NQ.-...QL.......E......H....
E. lutris                .D..T......PDPVQ.SF....H...I..T.I.P......P...S.........I.......---......NQ.-...QL.......E......H....
C. ursinus               .D..T......PDPTQ.SF....H...I..T.I.P......P...S.....H...I.......---.S....NQ.-...QL.......E......F....
Z. californianus         .D..T......PDPDQ.SF....H...I..T.I.P......P...S.....H...I.......---.S....NQ.-...QL.......E......F....
O. rosmarus              .D..T......PDPAQ.SF....H...I..T...P......P...S.....H...I.......---.S....NQ.-...QL.......E......F....
A. melanoleuca           .D..T......PDAAQ.SF....H...I..T.I.P......P...S.........I.......---......NQ.-...QL.......E......F....
U. maritimus             .D..I......PDAAQ.SF....H...I..T.I.P......P...S.........I.......---.....DNQ.-...QL.......E......F....
C. lupus                 ....T......PDPAQ.S.........I....T.P......P...S.........I.......---......NQ.-D..QL.......E...........
V. vulpes                ....T......PDPAQ.S.........I....T.P......P...S.........I.......---......NQ.-D..QL.......E...........
A. jubatus               ...........PD--Q.SF....H...I..I.I.P......P...S.................---......YQ.-....L..R....E...........
F. catus                 ....T......PD--Q.SF....H...I..I.I.P......P...S.................---....V.YQ.-....L..R....E...........
P. pardus                ....T......PD--Q.SF....H...I..I.I.P......P...S.................---....V.YQ.-....L..R....E...........
E. asinus                ...........PD--QS.I........I....I.P..........S.................---...AS..Q.-....L.......E...........
E. caballus              ...........PD--QS.L........I....I.P..........S.................---...AS..Q.-....L.......E...........
C. simum                 .D.........PG--QS.I........I....I.P..........S.................---....S..Q.-....LI......E......F....
B. taurus                ..........FPG--Q.EI....K...I....IAP..........S.......F.........---...APD.QP-...DFL.H....E...........
B. bubalis               ..........FPG--Q.EI....K...I....IAP..........S.......F.........---...APD.QP-...DFL.H....E...........
P. hodgsonii             .....R....FPG--Q.EI....K...I....IAP..........S.......F.........---...APD.QP-...DFL.N....E...........
C. hircus                ..........FPG--Q.EI....K...I....IAP..........S.......F.........---...APD.QP-...DFL.N....E...........
S. scrofa                .....N.....PS--Q...........I.....AP......P...........F.........---....A..Q-H....LH......E......F....
C. dromedarius           ...........PG--Q..I........I......P..........S.......FRI.......---.......E.-....L.......E...........
C. ferus                 ...........PG--Q..I........I......P..........S.......FRI.......---.......E.-....L.......E...........
V. pacos                 ...........PG--Q..I........I......P..........S.........I.......---.....A.E.-D...L.......E...........
O. orca                  ...........LG--Q.EI....K...IG...MSP..........S.....R.F.L....D..---...AP.QQ.QQ.EDLL.H....E...........
L. obliquidens           ...........LG--Q.EI....K...IG....SP..........S.....R.F.L....D..---....P.QQ.QQ.EDLL.H....E...........
T. truncatus             ...........LG--Q.EI....K...IG....SP..........S.....R.F.L....D..---....P.QQ---.EDLL.H....E...........
G. melas                 ...........LG--Q.EI....K...IG....SP..........S.....R.F.L....D..---....P.QQ.Q..EDLL.H....E...........
N. asiaeorientalis       ...........LG--Q.EI....K...I....MSP..........S.......F.L....D..---...TP.QQ.QQ.EDLL.H....E...........
P. sinus                 ...........LG--Q.EI....K...I....MSP..........S.......F.L....D..---...TP.QQ.QQ.EDLL.H....E...........
M. monoceros             ...........LG--Q.EI....K...IG...MSP......A...S.......F.L....D..---...AP.QQ.QQ.EDLL.H....E...........
D. leucas                ...........LG--Q.EI....K...IG...MSP......A...S.......F.L....D..---...AP.QQ.QQ.EDLL.H....E...........
L. vexillifer            ...........PG--Q.EI....K...I..I.ISP..........S.......F.L....D..---...AP.QR.QQQEDLL.H....E...........
P. catodon               ...........PG--Q.EI....K...I....ISP................R.F.S....D..---...AP.QQREWQEDVR.H....E..R........
B. musculus              ...........PG--Q.EI....K...I....I.P..........S.....R.F.L....D..---...AP.QQ.Q---DLR.H....E...........
B. acutorostrata         ...........PG--Q.EI....K...I....I.P..........S.....R.F.L....D..---...AP.QQ.Q---DLR.H....E...........
L. africana              ........D..LD--Q.VII.K.....T......P..........S........R........---.G..S..Q.-...Q...............F....
T. manatus               ........E..PD--Q.PII.K.....I....L.P..........S........R...I....---.G..S.YQ.-...Q........E......F....
E. edwardii              ........EA.PD--QDSFI.E.....I......P..................FR........---.G..S..Q.-.YE-L...N...E.....HF....
E. telfairi              DA.......WNLD--Q...F..L....I..T.I.P....-------------------------------------------------------------
C. asiatica              ...........PD--....I.......I..E.I.P..........S.......FR........---.G..S..Q.-....S......LE......F....

                                  410       420       430       440       450       460       470       480       490       500
                         ....|....|....|....|....|....|....|....|....|....|....|....|....|....|....|....|....|....|....|....|
H. sapiens               DRQTKQQPRQNVAYNREEERRRRVSHDPFAQQRPYENFQNTEGKGTAYSSAASHGNAVHQPSGLTSQPQVLYQNNGLYS--------SHGFGTRPLDPGT
P. troglodytes           ........................................M......................................--------.............
P. paniscus              ...............................................................................--------.............
P. anubis                ...M......................................V...............P.....S.....P.R..V...--------.............
M. mulatta               ...M......................................V...............P.....S.....P.R..V...--------.............
M. fascicularis          ...M......................................V...............P.....S.....P.R..V...--------.............
R. roxellana             ...M.....................................KV........T..................P.R..V...--------.............
C. capucinus             ..........S.........K.........H...R..........I...G.T......Y........L..P.....S.G--------LR...A...AQ..
A. nancymaae             ..............................H...H..........I...G.TG.....Y........L..P.......N--------L.........RE.
S. boliviensis           ..............................H...R..........I.S.G.T.P....Y.....A..V..P....A..G--------LS........R..
R. norvegicus            EK...S...L.E........K............VH..VKSAGA..LP.P.TT.....A..L..PA..IK.PLW...V.N--------H....A-------
M. musculus              EK...P.....E........K............AR..IKSAGAR.HSDP.TT.R.I..Q.L.WPAT.T---VW.....N--------Q.....-------
H. glaber                ...............M...............P..A.SA...RV.SP..P..VI.S..G...T.VA.....P.WS.EF.N--------LS.R.I.......
F. damarensis            .K.M....I......I...............P..F.SA...RV..P..A..-..S..G...M.V....HIP.WS.EF.N--------L..H.I.......
O. degus                 .Q......I.S..H.I...............P..F.SA...NV.DP..H...G.S.TG...V......HIQNWGREF.N--------L.RP.I..F..A.
C. lanigera              .Q......I.T....M...............P....ST...RL..L.SC.....S..G...P......HGP.W.SEFHN--------L.VP.........
C. porcellus             .K.....ST.HM...I...............P..N.SA...RV..P..P...NYS..S...T..S...H.PCWSSES.H--------V.R..A......I
I. tridecemlineatus      .K..................K.........H.N....V...R...L..PN....SS..N..T..N....GP.W.TD..T--------VP...V.....V.
M. marmota               .K..................K.........H.N....V...S...L..PN....SS..N..T.......GP.W.TD..N--------VP...I.....V.
O. cuniculus cuniculus   .K..R.P...S...S..........Y......T....V.K.GI.VHS.PGTT..S..A...T.......LP.W...V..--------L........E...
O. cuniculus algirus     .K..R.P...S...S..........Y......T....V.K.GI.VHS.PGTT..S..A...T.......LP.W...V..--------L........E...
S. floridanus            .K.MR.P...S...S..........Y......T....V.K.GI.VHS..GTT.PS..A...T.......PP.W...V..--------L........E...
S. bachmanni             .K.MR.P...S...S..........Y....P.T....V.K.GI.VHS.PGTT..S..A...T.......PP.W...V..--------L........E...
L. americanus            .K.MR.P...S...S..........Y....P.T....V.K.GI.VHS.PGTT..S..A...T.......PP.W...V..--------L........E...
L. timidus               .K.MR.P...S...S..........Y......T....V.K.GI.VHS.PGTT..S..A...T.......PP.W...V..--------L........E...
L. europaeus             .K.MR.P...S...S..........Y......T....V.K.GI.VHS.PGTT..S..A...T.......AP.W...V..--------L........E...
L. granatensis           .K.MR.P...S...S..........Y......T....V.K.GI.VHS.PGTT..S..A...T.......PP.W...V..--------L........E...
O. princeps              .K.MR.....S...S.................T.H..I..PGLQVPS.LG.VNQCSVL...VR.N..SPPP.W...F..--------L..L........A
O. collaris              .K.MR.....S...S.................T.H..I..PGLQVPS.LG.VNQCSVL...VR.N..SPPP.W...F..--------L..L........A
M. brandtii              .K........G....................P....TV..PGM..L.S.NT..P.......A...C.S.G.HWT--P.N--------PYD.RA...GA..
```

```
E. fuscus                 .K........G....................P....AV..PGV..L.S.NT..P.S.....A...C.SPG.HWT--P.N--------PYD.RA...GA..
M. natalensis             ...........MV..................P..H.TV.IPGV..L.A.N...PR.T...AV..A.....APPW..P.N--------PYDLR.GS.GA..
D. rotundus               ......P........................P...Q----LGV.DL.S.N.T....E....V.....T.G.HW...S..--------P...R..S.GL..
H. armiger_               ..............S................P.....V..PGI..L.ST..TT........T..........W..A.FG--------T...R.....LQ.
P. alecto                 .G.............................PK....V..PRI..L.S...T.........T........V.W...S..--------P.........L..
R. aegyptiacus            .G.....S.K.M..D................PK....V..PGI..L.S...T.........T.........NW...S..--------P.........L..
M. putorius               .....-PL....I..................PK....V..PGV-.LP....T.P...L...T.P........W....NN--------PFS.....Q.L.A
E. lutris                 .....-PL....V..................PK....V..PGV-.LT....T.P...L...T.P........W.....N--------PFP.....Q.L.A
C. ursinus                .....-PLK...........................HV..PGV..LP....T.P...L...T..........W.....N--------PFA.....Q.L..
Z. californianus          .....-PLK...........................HV..PGV..LP....T.P...L...T..........W.....N--------PFA.....Q.L..
O. rosmarus               .....-PLK...........................HV..PGV..LP....T.P...L...T..........W.....N--------PFA..S..Q.L..
A. melanoleuca            .....-PL..........................H..V..PGV..LP....T.P.S.L..ST..........W.....N--------PFT.....Q.L..
U. maritimus              .....-PL..........................H..V..PGV..LP......P...L..ST..........W.....N--------PFT.....Q.L..
C. lupus                  .....-PL..S..........................I..PGV..LP..I.T.P.......K......P.R.W..E...--------PFN.R....AL..
V. vulpes                 .....-PL..S..........................I..PGV..LP..I.T.P.......T......P.R.W..E...--------PFK.R....AL..
A. jubatus                .....-PL...MV...................K....V..PGV..IP....TG........T..........W.....N--------LY.L.A....L..
F. catus                  .....-PL...MV...................K....V..PGV..IP....TG........T..........W.....N--------LY.L.A....L..
P. pardus                 .....-PL...M....................K....V..PGV..IP....TG........T..........W.....N--------LY.L.A....L..
E. asinus                 ...................................D.V..SGI..LT.P..N..A......T......H.PHWH..S..--------P.........L..
E. caballus               ...................................D.V..SGI..LTHP..N..A......T......H.S.GH..S..--------P.........L..
C. simum                  .K.........M.......................D.V..SGI..L...G.N.Y.......A......H.P.W...S..--------P..L......L..
B. taurus                 ..R..E...P.M..SS.....................A.SPGI..L..PGVM..TS.AQ..A..S....SP.WRD.SF----------.PL.V....L..
B. bubalis                ..R..E...P.M..SS....K................A.SPGI..L..PGVM..TS.AQ..A..S....SP.WR..SF----------.PL.V....L..
P. hodgsonii              ..R..E...P.MT.SS...G.................A.SPGV..FT.PGVM..TS.AQ..A..S....SP.WRDVSFP--------LA.L.V....L..
C. hircus                 ..R..E...P.MTHSS.....................A.SPGI..F..PGVI..TS.AQ..A..S....SP.WRDASFP--------LP.L.V....L..
S. scrofa                 ..R..E..GGSA..GG..................H.PA.SPAV..L.CAG---P.S.AQH.A..S.H..P...SH.SHK--------PQ.L.A....L.A
C. dromedarius            ..R..E.....A..S....K................AA..PGI..P.C.G.T.....T...A......RL..WS..SC.--------P.........L.A
C. ferus                  ..R..E.....A..S....K................AA..PGI..P.C.G.T.....T...A..I...RL..WS..SC.--------P.........L.A
V. pacos                  ..R..E.........S....K...............AA..PGI..PTC.G.T..S..T...A......HL..WS..SC.--------P.........L.A
O. orca                   ..H.EE.......DR.Q....................A..AGL..L...G..N..S..Q..A.......LPHWR...FT--------.L...A..V.LR.
L. obliquidens            ..H.EE.......DR.Q....................A..PGL..L...G.TN..S..Q..A.......LPHWR...CT--------.L...A..V.LR.
T. truncatus              ..H.EE.......DR.Q....................A..PGL..L...G.TN..S..Q..A..A....LPHWR...CT--------.L...A..V.LR.
G. melas                  ..H.EE.......DR.Q....................A..PGL..L...G.TN..SV.Q..A.......LPHWR...CT--------.L...A..V.LR.
N. asiaeorientalis        ..H.EE.......DR.Q...............G....A..PGL..L...G.T...S..Q..A.......L..WR...CT--------.L...A..V.LR.
P. sinus                  ..H.EE.......DR.Q...............G....A..PGL..L...G.T...S..Q..A.......L..WR...CT--------.L...A..V.LR.
M. monoceros              ..H.EE.......DR.Q....................A..PGL..L...G.TN..S..Q..A.......L.HWR...C.--------.L...A..V.LR.
D. leucas                 ..H.EE.......DR.Q....................A..PGL..L...G.TN..S..Q..A.......L.HWR...CT--------.L...A..V.LR.
L. vexillifer             ..R.EE.......DR.Q....................A.SPGL..L...G..N.....Q..T.......L..YR...CT--------.L...A..V.LRN
P. catodon                ..H.EER.GWD..DSGQ.................C.DARRPGL..L.NPG..H..G..Q..A.......L..WR...CP--------.P.L.A..V.LRA
B. musculus               .GH.GG..GR...DS.Q.................S..A..PGL..L...G........Q..T.......L.SWR...CT--------PL...AS.V.LR.
B. acutorostrata          .GH.GR..GR...DS.Q....................A..PGL..L...G........Q..T.......L.SWR...CT--------PL...AS.V.LR.
L. africana               ..KE.W..............K................T..PGV..I..P..T..VDVFQ.LP.......LP.W.S....SS------P..S.A.T....I
T. manatus                ..KE.W..............K................T..PGV..L..PT.T..TDVFP.L.........P.W.....NS-------P..S.A......I
E. edwardii               ..KE.W----..........K..........P.L...A..PGM..L.S....N.SDR.LHTP.......IT.W.S..HNNQYNSYTTVNQ--I.TP..R.
E. telfairi               ----------------------------------------------------------------------------------------------------
C. asiatica               ..TV.G.......F......K..........P.T...ELKPGV..P....TTC..D.FQ.QP.P.N....P.W..R..NH---------P.TLGTRLHDP

                                   510       520       530       540       550       560       570       580       590       600
                          ....|....|....|....|....|....|....|....|....|....|....|....|....|....|....|....|....|....|....|....|
H. sapiens                AGPRVWYRPIPSHMPSLHNIPVPETNYLGNTPTMPFSSLPP-TDESIKY--TIYNSTGIQIGAYNYMEIGGTSSSLLDSTNTNFKEEPAAKYQAIFDNTT
P. troglodytes            .........................................-.......--.....................................S...........
P. paniscus               ....................................G....-.......--.................................................
P. anubis                 .......G....P.S..YK........V........T...L-.......--A................V............M.......S..........
M. mulatta                .......G....P.S..YK.................T...L-.......--A................V....................S..........
M. fascicularis           .......G....P.S..YK.................T...L-.......--A................V....................S..........
R. roxellana              .D.....GLL..P.S...K........V........T...L-....V..--A................V....................S..........
C. capucinus              .......G.....P...YKS......F.............L-P.....C--..F..S...........V..M....S.N..V.......S...D......
A. nancymaae              .......G....L....YKS......F............S.-P.....C--..F..S...........V..M....S....M.......S...D......
S. boliviensis            .......G....L...QYKS......F..............-PE....C--..F..R...........V..M....S....V.......S...D......
R. norvegicus             T.TG...G.SV.QSYNAYKT......LP.SI....YI..A.-P.D..RC--.....SRV...N....DL-.P..QPPTN.C---.V.STSRH..V.A...
M. musculus               T.TG...P.NL.Q.Y.TYKT......IP.S.....YF.G.V-A.DL...--..F..S.....NH...DV-.LN.QPPNN.C---...STSRH........
H. glaber                 P.SGI..ALH..Q..N.FKT......PA.S...L..Y...S-...LM.C--N.S........T........MN.Q.DNV-C..LE..S.S..........
F. damarensis             S.--I..ALH..QI...FKT....SSGA.S...L..Y...S-...LM.C--N.S........T........M..Q.-.N----LE..S.S..........
O. degus                  P.AGTC.G.H..Q.A..FKT......LA.S...L.LFCFSS-...L..SQVNVS..SN..F........RRMN.QP-EN.C..LE..STS..........
C. lanigera               S.AGI..G.HS.Q.SN.SKT......LA.....L..Y.FIS-P..L..SHIN.S..S...F........TPVN.Q.-EN.R..LE..S.S......A...
C. porcellus              PST-..FGSHLNQ..NPFTP.M...SLA.S...L.LY.WM.-P..LLRSHIN.S......F.TS.....S.MNAP.-EN.C..VE..S.S..........
I. tridecemlineatus       .STGF..GLNS.P..N.YKT......LPA....I.LY.F.S-....P.C--C..........T.....V.AVN.Q.PEN.CA.V...LS.FRD......S
M. marmota                .STGF..GLNS...SN.YKT......LPAS...I.LY.F.S-....P.C--C................V.AVN.Q.PENICA.V...LS.FRD......S
O. cuniculus cuniculus    ..QA...G.SL.P.S..YKT......L......I.....S.-AE..M.C--S....S...L..C.......I..PP.E-CLCTNLQ...S.......D..
O. cuniculus algirus      ..QA...G.GL.P.S..YKT......L......I.....S.-AE..M.C--.....S...L..C.......M..PP.E-CLCTNLQ...S.......D..
S. floridanus             T.QPI..G.SL.P.S..YKT....S.L......I.....L.-PE..V.C--.....S...L..C.......M..-P..-CSCTGLQ...S.......D..
S. bachmanni              T.QT...G.SL.P.S..YKT......L......I.....S.-AE.AM.C--S....S...L..C.....S.V..-P..-CSCTGLQ...S.......D..
L. americanus             T.QT...G.SL.P.S..YKT......L......I.....S.-AE.AM.C--S....S...L..C.......M...P.E-CSCTHLQ...S.......D..
L. timidus                T.QT...G.SL.P.S..YKT......L......I.....S.-AE.AM.C--S....S...L..C.......M...P.E-CSCTHLQ...S.......D..
L. europaeus              ..QT...G.SL.P.S..YKT......L......I.....S.-AE.AM.C--S....S...L..C.......M...P.E-CSCTHLQ...S.......D..
L. granatensis            ..QT...G.SL.P.S..YKT......L......I.....S.-AE.AM.C--S....S...L..C.......M...P.E-CSCTHLQ...S.......D..
O. princeps               V..S...ASAG.QIQ.Q.KT......L......I..T..A.-AE....C--.....S......C.......GL.P.S.-GLYANPQ..PL.DH....DA.
O. collaris               V..S...ASAG.QIQ.Q.KT......L......I..T..A.-AE....C--.....S......C.......GL.P.S.-GLYANPQ..PL.DH....DA.
M. brandtii               IDS.F.SG.NA.QI....KT......L......I..I..TS-R..TA..--A....A.V...DN.R..V-.V.LP.....YV.WN-...S...DT.....
E. fuscus                 G.S.F.SG.DANQ..G..KT.....TL......I..I..TS-R..TA.C--A....A.V...DN.R..V-.VNLP.....YV.W.-...S...Y......
M. natalensis             ....F..G.NA..T.G..KT......L.E....I..I.RTS-RG.TAQ.--S....A.V...DS.H..V..M.LPFP.Y.YM.W.-...SA..D......
D. rotundus               V.S..CCG.N........KT......L......I..V..SS-.G..V.C--S..........DK....V..V..P.....YI.L.-...S...D......
H. armiger_               V.S..CNG.N..Q.S..PKT......LP.....I..V.F.S-R...VRC--S......V...DN.H....RMN.WS....YM.L.....S..LD..G...
P. alecto                 V.S....G.T........KN......L...L..I..I...S-R...V.C--S....A.....DN....V.ER..L.....Y..S...SIS..LD..G...
R. aegyptiacus            V.S....G.T........KS......L...L..I..I..SS-G...V..--S....A.V...DN....V.....P.P.N.Y..S....IL..LD..G...
M. putorius               .SS.A..G.N.N....V.KT......L....A.LS.G.F.S-R...V.C--S.R..A.....DH.....S.M..VP..---M..........HD..E.I.
E. lutris                 .SS.A..G.N......V.KT......L......LS.ATF..-R...V.C--S.H........EH.....S.M..VP..---M.L........HD..E.I.
C. ursinus                T.S..L.G.N......V.K.......L......L.C..F.S-R...V.C--.....A...V.DH.....S.M..V.P.---M.L.....S...D..E...
```

```
Z. californianus        T.S..L.G.N......V.K.......L......L.C..F.S-R...V.C--.....A...V.DH.....S.M..V.P.---M.L.....S...D..E...
O. rosmarus             T.S..L.G.N......V.K.......L......L....F.S-R...V.C--.....A...V.DH.....S.M..V.P.---M.L.....S...D..E.I.
A. melanoleuca          ..S....G.N......V.KT......L......I..T.F.S-R...V.C--A....AS..V.DH.......MN.V.P.---M.L.....S...D..E.V.
U. maritimus            ..S....G.N......V.KT......L......I..T.F.S-R...V.C--A....A...V.DH.......MN.V.P.---M.L.....S...D..E.V.
C. lupus                T.S....Q.N...I..VNKT......L......I....F.S-R...V.C--.....S...V.EH....V..M..A...---M.V.....Y...D....V.
V. vulpes               T.S....Q.N...I..VNKT......L......I....F.S-R.....C--.....S...V.EH....V..M..A...---M.V.....Y...D....V.
A. jubatus              PS.....G.N......V.KT......L......I......S-R.....C--N....A...V.DH.......LN.A..E..HV.L.....S...D....I.
F. catus                PS.....G.N......V.KT......L......I......S-R.....C--N....A...V.DH.......LN.A..E..HV.L.....S...D....V.
P. pardus               PS.....G.N......V.KT......L......I......S-R.....C--N....A...V.DH.......LN.A..E..HV.L.....S...D....I.
E. asinus               ....A..GAN....S.M.KT......L......I..I.F.S-R...A..--...........D........MT.Q...G.YM.L.Q...S...D....M.
E. caballus             ....AR.GAN....S.M.KT......L......I..I.F.S-R...A..--..C........H........MT.Q...G.YM.L.Q...S...D....M.
C. simum                ..Q....V.N....S.V.KT......V......V..I.F.S-R...A.C--...........DN.......M..Q.....YM.L.....S...D......
B. taurus               MS.....G.N.G.....YKT......L......I..T...S-R...S..--..HS.S.....DS.......M...V....YM.L.....S..........
B. bubalis              MS.....G.N.G.....YKT......L......I..T...S-R...S..--..HS.S.....DS.......M...V....YM.L.....S..........
P. hodgsonii            TS.....T.N.G.....YKT......L..S...I..T.V.S-R...A..--..H..S.....DS.......M...V...MYM.L.....S.......T..
C. hircus               TSS....A.N.G.....YKT......L......I..T.V.S-R...A..--..H..S.....DS.......M...V...MYM.L.....S.......T..
S. scrofa               .DR....M.SQG.R...YKT.....DL......V..A...S-R.A.PR.--IL.GNS.V...DH....L.A.V.V....MCPHV.....S......E...
C. dromedarius          .........N.G.....YKT......L......I......S-R...V..--..HS.S.....DH......A....V...MFV.V.....S...D..G.S.
C. ferus                .........N.G.....YKT......L......I......S-R...V..--..HS.S.....DH......A....V...MFV.V.....S...D..G.S.
V. pacos                .........N.G.....YKT......L......I......S-R...V..--..HG.S.V...DH......A....V...MFM.V.....S...D..G.SA
O. orca                 ...G.GDG.N.G.....YKT......L...........F.S-R...L..--G....F.V...NN.F.....M.PLV....YM.L..D..S..RD......
L. obliquidens          ...G..DG.N.G.....YKT......L......I....F.S-R...L..--G...CS.....NN.F.....M.PLV....YM.L..D..S..RD...D..
T. truncatus            ...G..DG.N.G.....YKT......L......I....F.S-R...L..--G...CS.V...NN.F.....M.PLV....YM.L..D..S..RD......
G. melas                ...G..DG.N.G.....YKT......L......I....F.S-R...L..--G...CS.V...NN.F.....M.PLV....YM.L..D..S..RD......
N. asiaeorientalis      ...G..DG.N.G.....YKT......L....S.I....F.S-R...L..--G..KCS.V...NN.......M.PLV....YM.L.GD..S...D......
P. sinus                ...G..DG.N.G.....YKT......L.E..S.I....S.S-R...L..--S...CS.....NN.......M.PLV....YM.L.GD..S...D......
M. monoceros            ...G..DG.N.G.....YKT......L......I....F.S-R...L..--G....F.V...NN.......M.PLV....YM.L..D..S...D......
D. leucas               ...G..DG.N.G.....YKT......L......I....F.S-R...L..--G....F.V...NN.......M.PLV....YM.L..D..S...D......
L. vexillifer           T..G..NG.N.G....MYKT......L......I......S-RGK-------------------------------------------------------
P. catodon              ...G.CCG.H.G.T....KT.....HP......I......S-R...L..--S....S.V...KN.......MG.LV..G.YMSL.DA..SQ..D......
B. musculus             ...G...G.N.G.....YKT......L......I......S-R...L..--S....S.....NN.......M..VV....HM.L..G..S...D......
B. acutorostrata        ...G...G.N.G.....YKT......L......I......S-R...L..--S.C..S.....NN.......M..VV....HM.L..G..S...D......
L. africana             ....P..G.N.....N..KT......L......I...-F.S-I...FR.--S..........N.....V.RM.VPP.......L.....W...E....I.
T. manatus              ....P..G.N........KT.....SL.V....I..I.F.S-....FR.--N......V...N.....V.RM.VPPQ............S...E....I.
E. edwardii             S...P.FE.N...I.P..KT.....SL.SFHLPTIPFQSFSS....TR.--N.H..S.....NN.F.....AHTQP.E..SV.Y-....L...E..A.N.
E. telfairi             ------------------------------------------------------------------------------------------------..LS
C. asiatica             ETGGPGHWCN.TQV....KS....S.L......I....Y.S-.EN.VR.--N....S.....D.SF..V.RL.VPP....Y........S...E..G.V.

                                 610       620       630       640       650       660       670       680       690
                        ....|....|....|....|....|....|....|....|....|....|....|....|....|....|....|....|....|....|..
H. sapiens              SLTDKHLDPIRENLGKHWKNCARKLGFTQSQIDEIDHDYERDGLKEKVYQMLQKWVMREGIKGATVGKLAQALHQCSRIDLLSSLI-YVSQN
P. troglodytes          ......................................................................................-.....
P. paniscus             ......................................................................................-.....
P. anubis               .......................................................................V....A.....NH..-.....
M. mulatta              .......................................................................V....A.....NH..-.....
M. fascicularis         .......................................................................V....A.....NH..-.....
R. roxellana            ............................................................................A.....NY..-.....
C. capucinus            ................Q.........L............................L..........................NH.V-.I...
A. nancymaae            ................Q.......................................R.........................NH.V-.....
S. boliviensis          ................Q......................................L....V.............R.......NR.V-.....
R. norvegicus           .......N.....M..Q...........E..........................L....T...............C.T...NQ..-QA..S
M. musculus             ....E..N.......RQ...........E..........................L....T...............C.....NH..-RA..S
H. glaber               ....N..N.V......Q...........E...............H...H......L....S.........W..Y....K...NC..-HIT..
F. damarensis           ....S..T.V......Q...........E...............H...H......L....S.........S..Y....K...TG..-HT...
O. degus                ....NQ.NL.G.....Q..P........EP..............H...F......R....SQ........W..Y..F.K...NH.V-DRG.I
C. lanigera             ...ANQ.T.VE........P........E...............H...V......R....S.R.......W..Y....K..VNH.M-DI...
C. porcellus            .V..I..N.V......Q..T......I.E...............H....K.....E....N.........R..Y....K...NH..-DI.K.
I. tridecemlineatus     ...A...N.V..................E..........................L....N.....................TA..-H....
M. marmota              ...A...N.V..................E..........................L....N.....................NA..-H....
O. cuniculus cuniculus  .......S.V.D..............L.E..........................L....T.........R.......Q...TC..-QL..K
O. cuniculus algirus    .......S...D..............L.E..........................L....T.........R.......Q...TC..-QL..K
S. floridanus           .......S.V.D..............L.E..........................L....T.........R.......K...TC..-QL..K
S. bachmanni            .......S...D..............L.E..........................L....T.........H.......K...TC..-QL..K
L. americanus           .......S.V.D..............L.E..........................L....T.........R.......K...TC..-QL..K
L. timidus              .......S.V.D..............L.E..........................L....T.........R.......K...TC..-QL..K
L. europaeus            .......S.V.D..............L.E..........................L....T.........R.......K...TC..-QL..K
L. granatensis          .......S.V.D..............L.E..........................L....T.........R.......K...TC..-QL..K
O. princeps             ....T..S.V.D.......T........E..........................L....T.........R..Q.SA.....NY.V-RL..K
O. collaris             ....T..S.V.D.......T........E..........................L....T.........R..Q.SA.....NY.V-RL..K
M. brandtii             .........V.....RT...........E..........................L....S.........R..Y........N..V-FI.M.
E. fuscus               .........V.....RT...........E..........................L....S.........W..Y........NF.G-TI...
M. natalensis           ....E....V.....RT..S....M...E..........................L....S.R.......R..Y........NC.VAHI...
D. rotundus             .........V.....RS..S........E..........................L....N.........Y.......L...NA.K-.I..H
H. armiger_             .........V.D....S..S........E..........................L....N.........YS.Y.......VN...-HI...
P. alecto               .........V.....RS..I.......SE..........................L....N........TY..Y....T...NA.V-.I...
R. aegyptiacus          .........V.....RS..S......ISE..........................L....N.........Y..Y..A.T...NA.V-.I...
M. putorius             .........V......Q...........EP.........................Q....N.........Y...........NY.V-HI..S
E. lutris               .........V......Q...........EP.........................Q....N.........Y..Y....T...NY.V-RI..S
C. ursinus              .........V......Q..........SEP..E...................KT.L....N.........Y..Y........NY.V-HIN.S
Z. californianus        .........V......Q..........SEP..E...................KT.L....N.........Y..Y........NY.V-HIN.S
O. rosmarus             .........V......Q..........SEP..E...................KT.L....N.........Y..Y........NY.V-HIN.S
A. melanoleuca          .........V......Q...........EP.........D...............L....N.........Y..Y........NY.V-HI..S
U. maritimus            .........V......Q...........EP.........................L....N.........Y..Y........NY.V-HI..S
C. lupus                .........V......Q...........EP.........................L....N.........Y..Y........NY..-HI...
V. vulpes               .........V......Q...........EP.........................L....N.........Y..Y........NY..-HI...
A. jubatus              .........V.....RQ...........E..........................L....S.........Y..Y........NY..-HI..S
F. catus                .........V.....RQ...........E..........................L....S.........Y..Y........NY..-HI..S
P. pardus               .........V.....RQ...........E..........................L....S.........Y..Y........NY..-HI..S
```

```
E. asinus            .........V...............S..E........................R.L....N.........Y...........NH..-HI..Y
E. caballus          .........V...............S..E........................R.L....N.........Y...........NH..-HI..Y
C. simum             .........V...............N..E..........................L....N.........C..Y........HR..-HI...
B. taurus            .........V.....R...........SE..........................L..A.S.........C..YL.....VNC..-RI..S
B. bubalis           .........V.....R...........SE..........................L..A.S.........Y..YL.....VNC..-RI..S
P. hodgsonii         .........V.....R...........SE..........................L....S.........F..YL.....VNC..-RI...
C. hircus            .........V.....R...........SE..........................L....S.........L..YL.....VNC..-R....
S. scrofa            .........V......N..I......L.E..........................L....S.........R..Y....L....C..-RS...
C. dromedarius       .........V...............Y..E..........................L....N.........Y..Y.......VNY..-NI...
C. ferus             .........V...............Y..E..........................L....N.........Y..Y.......VNY..-NI...
V. pacos             .........V...............Y..E..........................L....N.........Y..Y.......VNH..-NI...
O. orca              .........V.....RQ..D.......S...........................L....S.........F..Y....L....H.T-HS...
L. obliquidens       .........V.....RQ..D.......S...........................L....S.........F..Y....L...RH.T-HS...
T. truncatus         .........V.....RQ..D.......S...........................L....S.........F..Y....L....H.T-HS...
G. melas             .........V.....RQ..D.......S...........................L....S.........F..Y....L...NH.T-HS...
N. asiaeorientalis   .........V.....RQ..D.......Y...........................L....S.........W..Y....V....H..-HS...
P. sinus             .........V.....RQ..D.......Y...........................L....S.........W..Y....V....H.T-HS...
M. monoceros         .........V.....RQ..D.......S...........................L....S.........W..Y....V....H.T-HS...
D. leucas            .........V.....RQ..D.......S...........................L....S.........W..Y....V....H.T-HS...
L. vexillifer        --------------------------------------------------------------------------------------------
P. catodon           .........V..........F......S...........................L....S.........C...R...T...NC..-HA..D
B. musculus          ....R....V..........F......S...........................L....S......R..Y..YE...V...NC..-CM...
B. acutorostrata     ....R....V..........F......S...........................L....S.........C..Y....HSDT.---------
L. africana          .........V.....R...K........EP.........................L....S.........Y..Y....T...NY..-R....
T. manatus           .........V.....R...K........EP.........................L....N.........Y..Y....T...NY.V-R....
E. edwardii          .........V......Q..K........EP..............R..........L.K..NR.......TY..YH......VNY.K-D....
E. telfairi          .........V.........K........EP.........................L....N.........H..Y......I.HY..-VA...
C. asiatica          .........V.........K........EP.........................L....S.........Y..Y.......VN...-V...H
```
