# Supplementary material for: Convergent Loss of the Necroptosis Pathway in Disparate Mammalian Lineages Shapes Viruses Countermeasures
Source: Front Immunol. 2021 Sep 1;12:747737. doi: 10.3389/fimmu.2021.747737 (PMC8445033; doi:10.3389/fimmu.2021.747737)

Supplementary file 7. Mutational validation of *MLKL* and *RIPK3* in Cetacea.

Supplementary file 7.1. Validation of a premature in-frame stop codon in the first coding exon of *MLKL* across representative species of the studied Cetacea families.

Tursiops truncatus (Delphinidae)

Blasted SRA BioProjects: PRJNA356464 and PRJNA20367

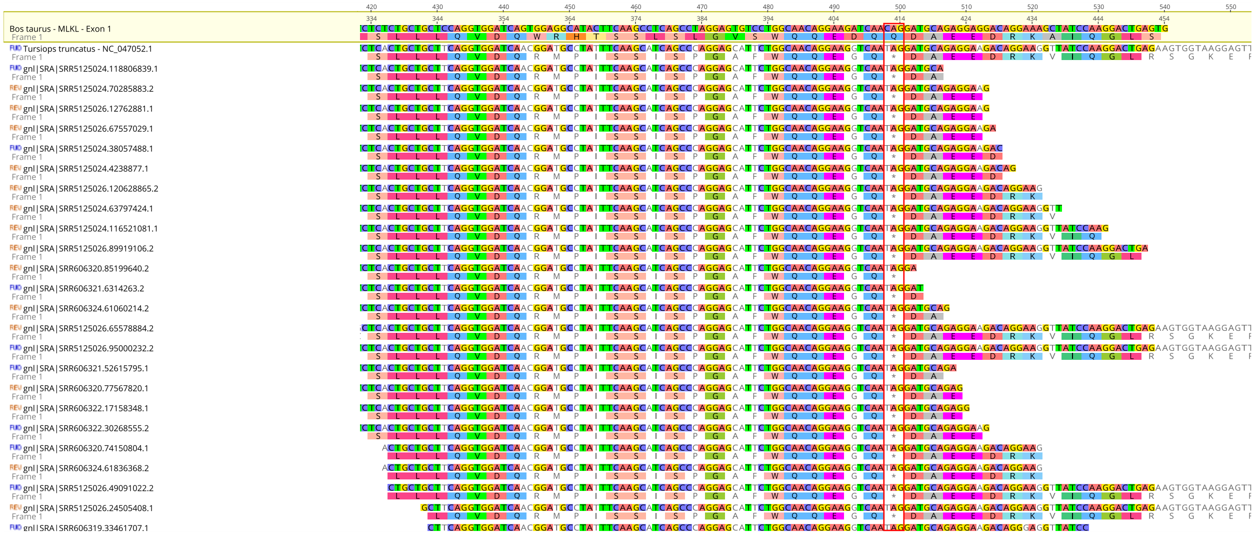

Neophocaena asiaeorientalis asiaeorientalis (Phocoenidae)

Blasted SRA BioProjects: PRJNA433603 and PRJNA371395

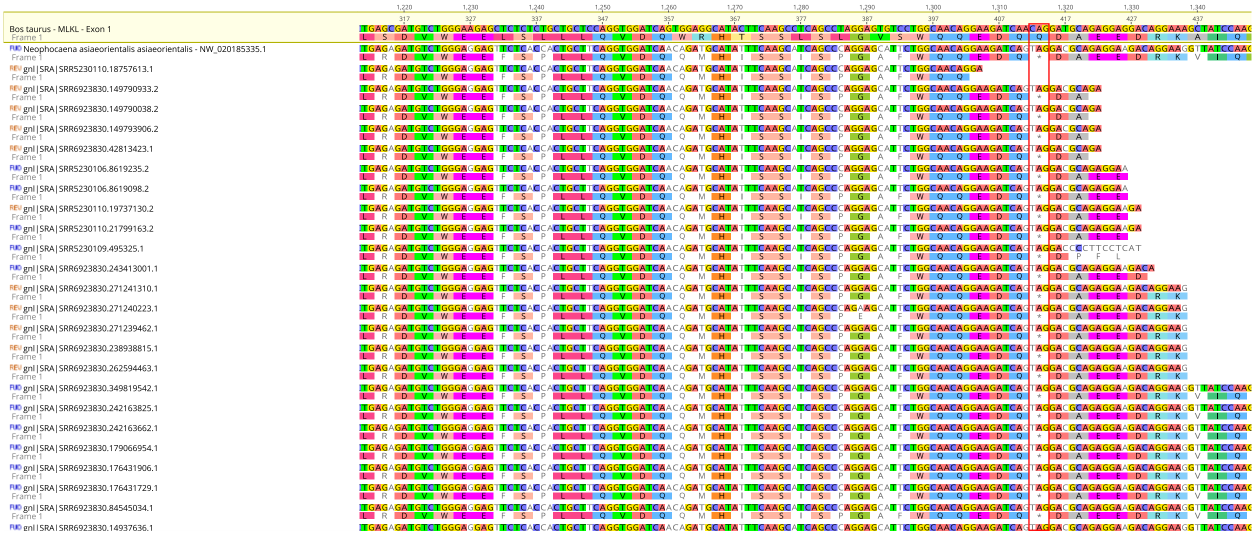

Monodon monoceros (Monodontidae)

Blasted SRA BioProjects: PRJNA520934 and PRJNA508363

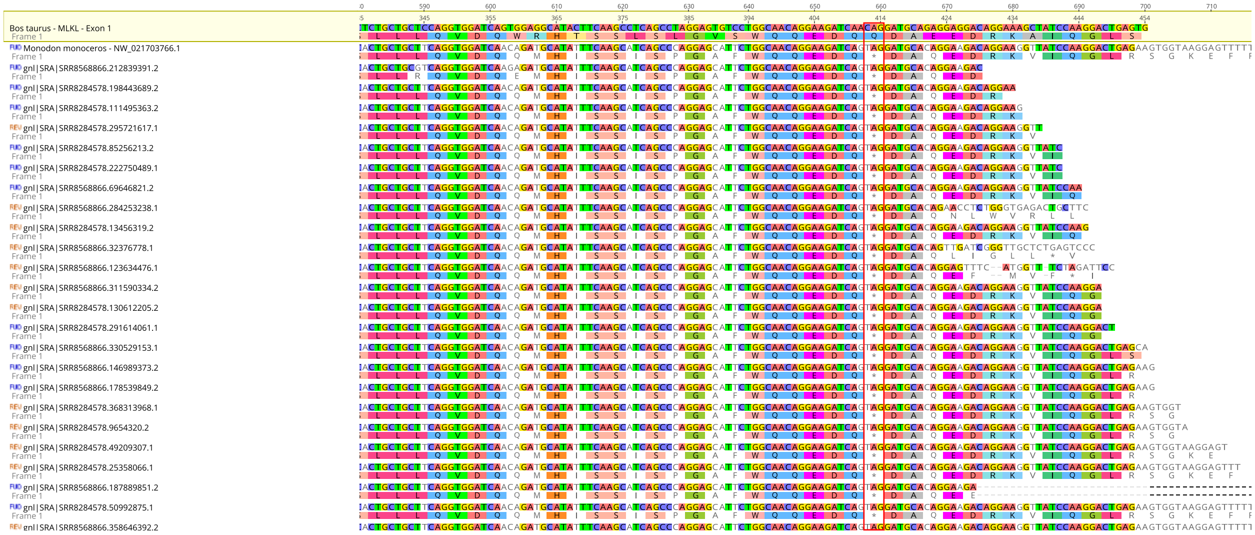

Physeter catodon (Phocoenidae)

Blasted SRA BioProjects: PRJNA89089 and PRJNA411766

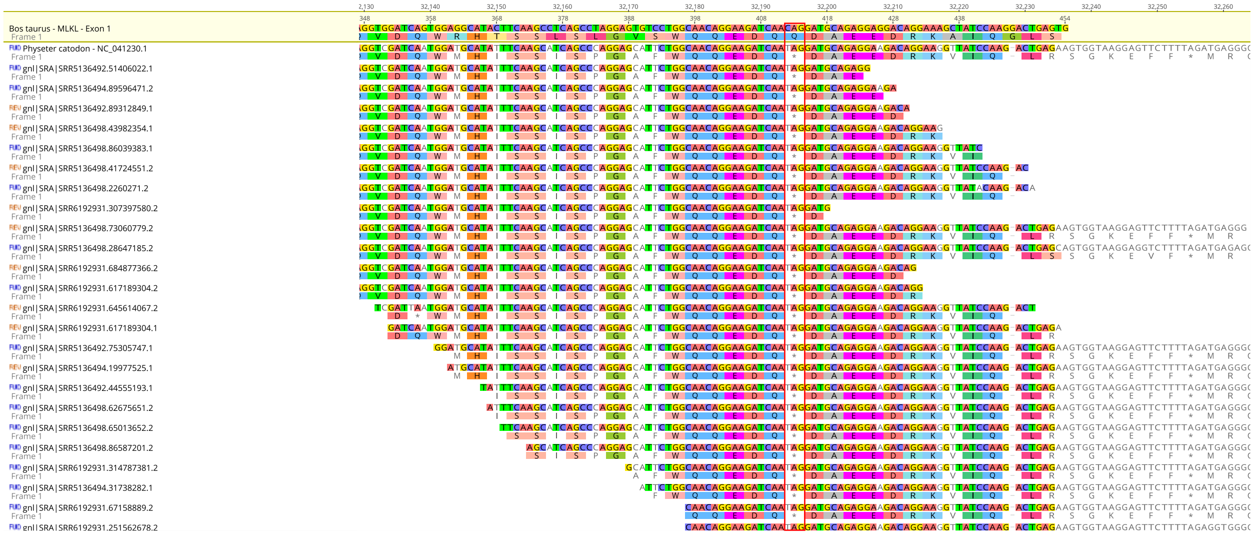

Balaenoptera acutorostrata scammoni (Balaenopteridae)

Blasted SRA BioProjects: PRJNA607895 and PRJNA335553

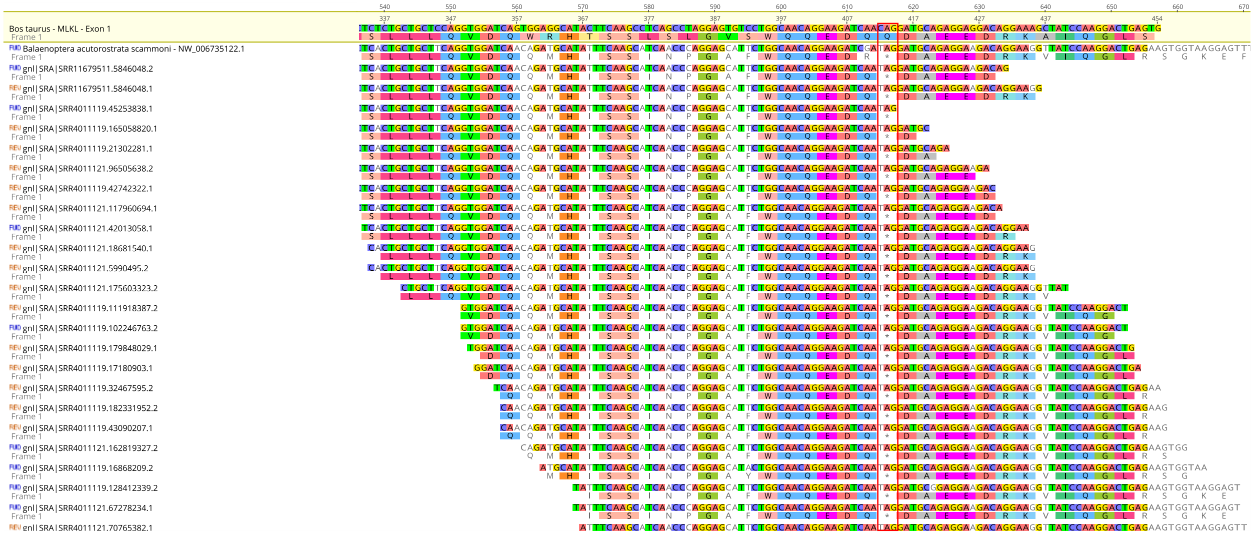

Supplementary file 7.2. Validation of a single-nucleotide deletion in the sixth coding exon of *RIPK3* across representative species of the studied Cetacea families.

Tursiops truncatus (Delphinidae)

Blasted SRA BioProjects: PRJNA356464 and PRJNA20367

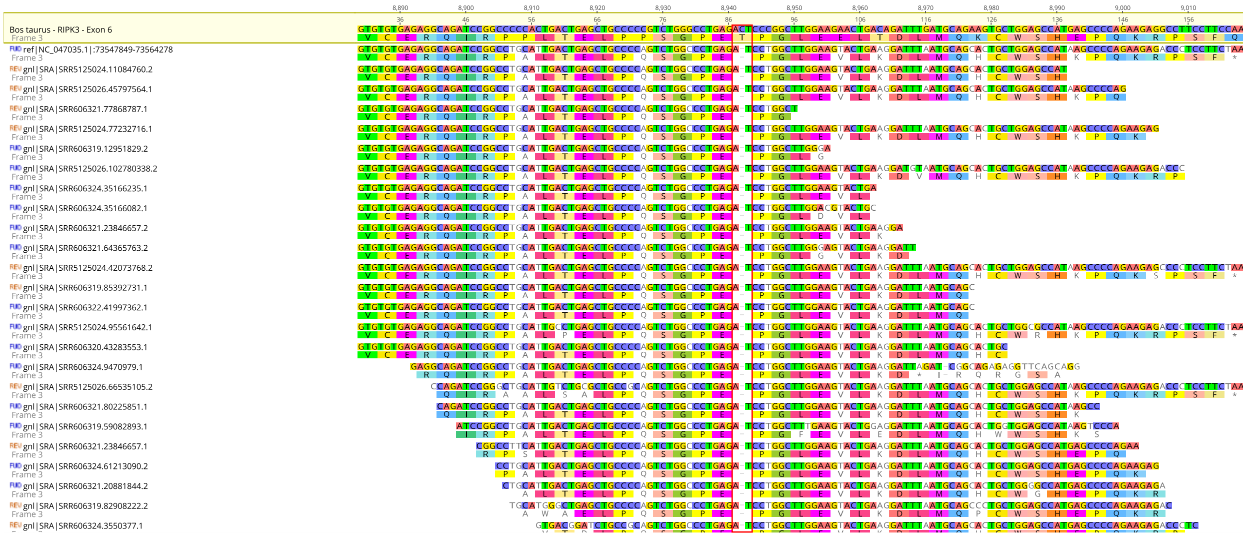

Neophocaena asiaeorientalis asiaeorientalis (Phocoenidae)

Blasted SRA BioProjects: PRJNA433603 and PRJNA371395

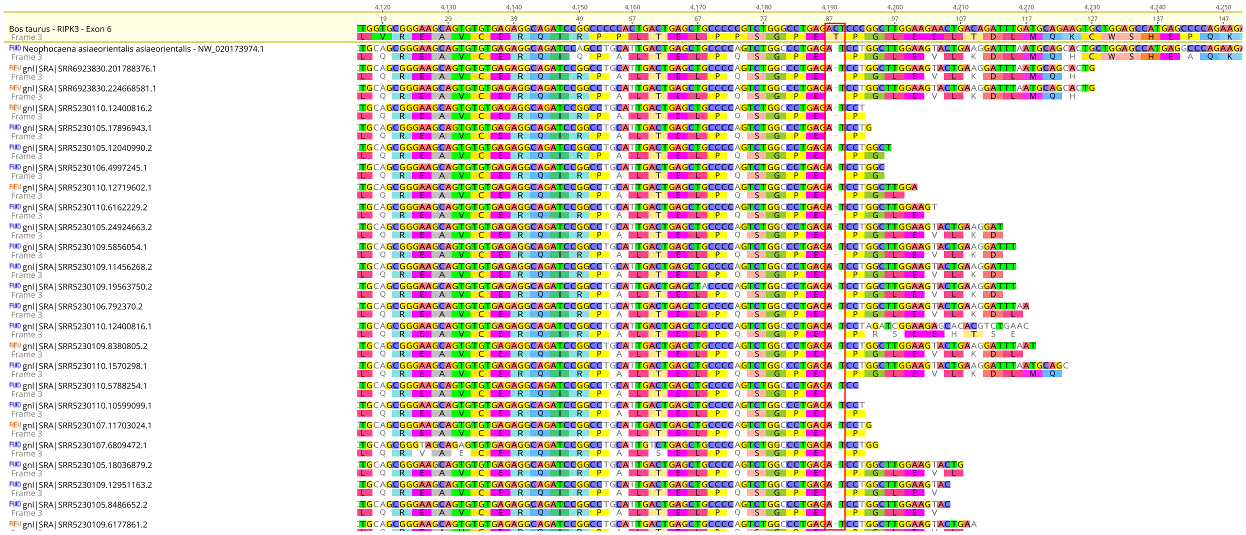

Monodon monoceros (Monodontidae)

Blasted SRA BioProjects: PRJNA520934 and PRJNA508363

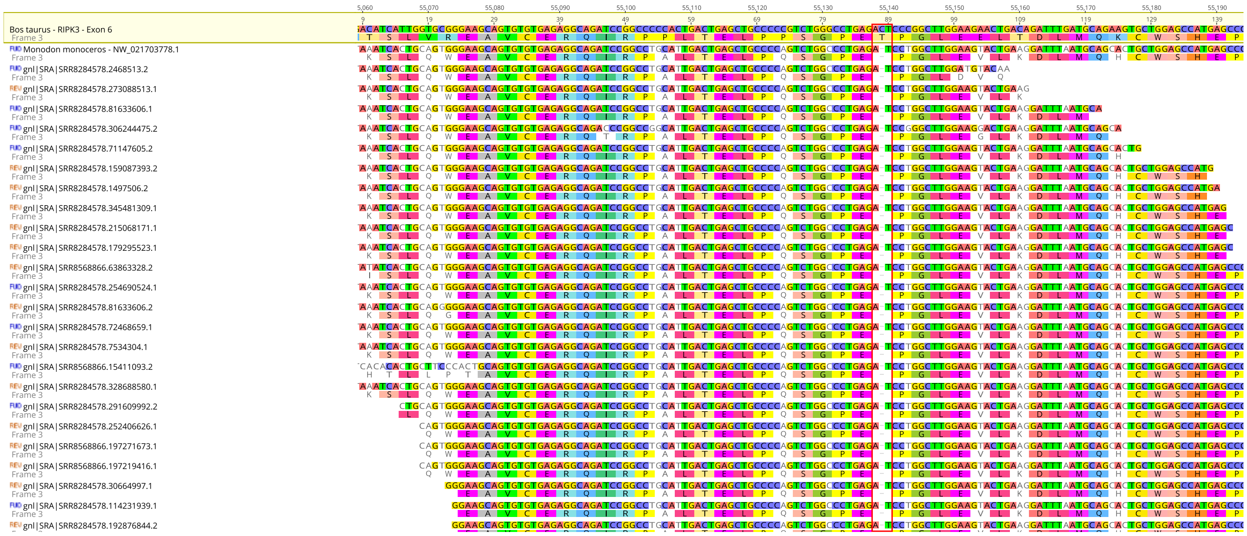

Physeter catodon (Phocoenidae)

Blasted SRA BioProjects: PRJNA89089 and PRJNA411766

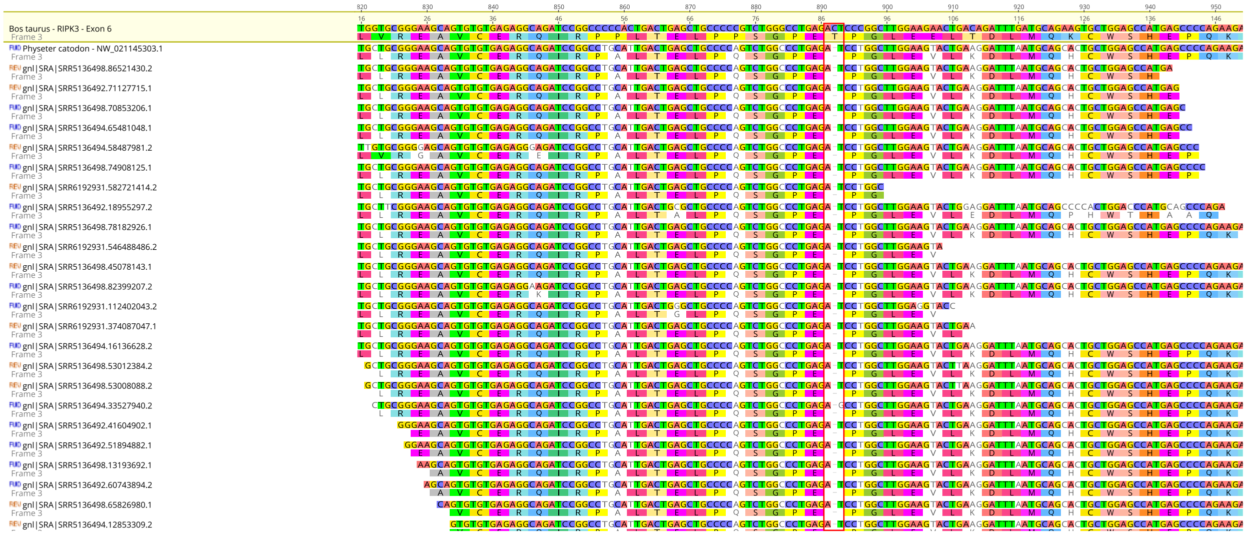

Balaenoptera acutorostrata scammoni (Balaenopteridae)

Blasted SRA BioProjects: PRJNA607895 and PRJNA335553

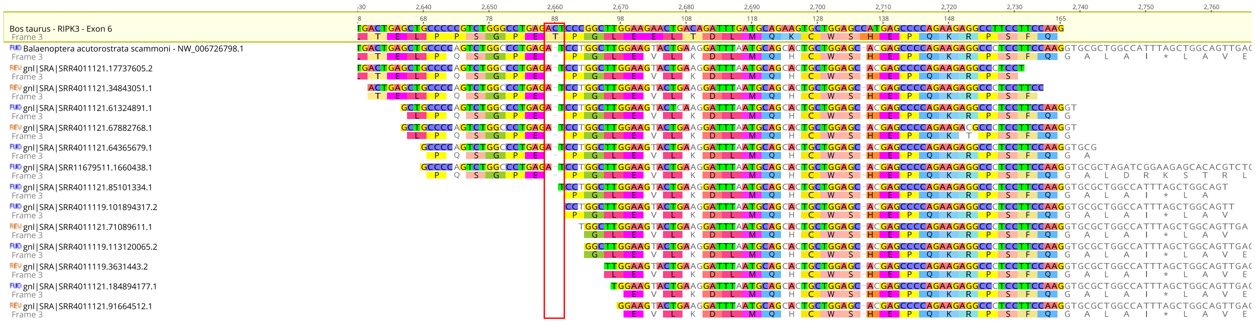

Supplement: Supplementary file 7 [file DataSheet_7.pdf]
